# Rhythmic transcription of *Bmal1* stabilizes the circadian timekeeping system in mammals

**DOI:** 10.1038/s41467-022-32326-9

**Published:** 2022-08-23

**Authors:** Yasuko O. Abe, Hikari Yoshitane, Dae Wook Kim, Satoshi Kawakami, Michinori Koebis, Kazuki Nakao, Atsu Aiba, Jae Kyoung Kim, Yoshitaka Fukada

**Affiliations:** 1 Department of Biological Sciences, School of Science, The University of Tokyo, Hongo 7-3-1, Bunkyo-ku, Tokyo, 113-0033 Japan; 2 Circadian Clock Project, Tokyo Metropolitan Institute of Medical Science, Kamikitazawa 2-1-6, Setagaya-ku, Tokyo, 156-8506 Japan; 3 Department of Mathematical Sciences, Korea Advanced Institute of Science and Technology, Daejeon, 34141 Republic of Korea; 4 Biomedical Mathematics Group, Institute for Basic Science, Daejeon, 34126 Republic of Korea; 5 Laboratory of Animal Resources, Center for Disease Biology and Integrative Medicine, Graduate School of Medicine, The University of Tokyo, Hongo 7-3-1, Bunkyo-ku, Tokyo, 113-0033 Japan; 6 Present Address: Department of Mathematics, University of Michigan, Ann Arbor, MI 48109 USA; 7 Present Address: Institute of Experimental Animal Sciences, Graduate School of Medicine, Osaka University, 2-2 Yamadaoka, Suita, Osaka, 565-0871 Japan

**Keywords:** Circadian rhythms, Oscillators

## Abstract

In mammals, the circadian clock consists of transcriptional and translational feedback loops through DNA *cis*-elements such as E-box and RRE. The E-box-mediated core feedback loop is interlocked with the RRE-mediated feedback loop, but biological significance of the RRE-mediated loop has been elusive. In this study, we established mutant cells and mice deficient for rhythmic transcription of *Bmal1* gene by deleting its upstream RRE elements and hence disrupted the RRE-mediated feedback loop. We observed apparently normal circadian rhythms in the mutant cells and mice, but a combination of mathematical modeling and experiments revealed that the circadian period and amplitude of the mutants were more susceptible to disturbance of CRY1 protein rhythm. Our findings demonstrate that the RRE-mediated feedback regulation of *Bmal1* underpins the E-box-mediated rhythm in cooperation with CRY1-dependent posttranslational regulation of BMAL1 protein, thereby conferring the perturbation-resistant oscillation and chronologically-organized output of the circadian clock.

## Abstract (web summary)

The mammalian circadian clock is composed of clock genes forming transcriptional feedback loops. Here, the authors identify a key role of the secondary feedback loop that is interlocked with the core loop to establish a perturbation-resilient clock system.

## Introduction

**Fig. 1:**
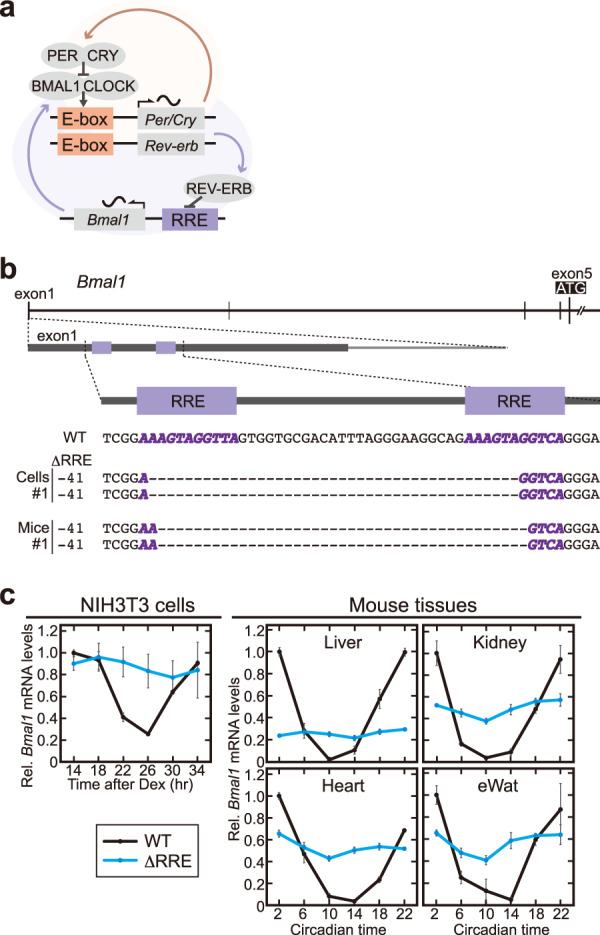
Two RREs in the 5’-UTR of *Bmal1* are essential for rhythmic expression of *Bmal1*. **a** A model for the mammalian circadian clock composed of two interlocking loops through DNA *cis*-elements, E-box and RRE. CLOCK and BMAL1 activate the E-box-mediated transcription of a series of genes such as *Per*, *Cry*, and *Rev-erb* genes, and the transactivation is suppressed by translated PER and CRY proteins. REV-ERB proteins repress the RRE-mediated transcription of a series of genes such as *Bmal1*. **b** The genome structure of the *Bmal1* gene locus (Upper panel) and the sequences of ΔRRE cell line #1 and ΔRRE mouse line #1 (Lower). Thick and thin lines indicate exons and introns of *Bmal1*, respectively. RRE elements are indicated by two boxes in purple (*Upper*) or bold italic letters in purple (Lower). **c** Temporal profiles of *Bmal1* mRNA levels. Fibroblasts (NIH3T3 cells), livers, kidneys, hearts and epididymal white adipose tissues (eWAT) were harvested at 4-hr intervals, followed by quantitative RT-PCR. The mRNA levels of *Bmal1* were normalized by *Rps29*. Data are means ± SEM (*n* = 3 for WT, *n* = 4 for ΔRRE). Blue and black lines indicate the ΔRRE mutants and the littermate controls, respectively.

The circadian clock regulates 24-hour rhythms of physiological functions in a variety of organisms^1,2^. In mammals, the circadian clock oscillation is based on transcriptional and translational feedback loops^1^, in which DNA *cis*-elements, E-box and RRE, play pivotal roles for regulating transcriptional rhythms of a series of clock genes^3^. A heterodimer of CLOCK and BMAL1 activates transcription of *Period* (*Per*) and *Cryptochrome* (*Cry*) genes through E-box, and the transactivation is suppressed by PER and CRY proteins (Fig. 1a). In addition to this negative feedback loop, transcription of *Bmal1* is known to be regulated by RRE in a circadian manner^3^. RRE-mediated transcription is activated by ROR (α/β/γ) proteins, and it is repressed by REV-ERB (α/β) whose transcription is regulated by E-box^4,5^. In this way, the E-box-mediated core loop is interlocked with the RRE-mediated additional loop (Fig. 1a), and hundreds of clock-controlled genes are rhythmically regulated by these *cis*-elements or combinations of several *cis*-elements. For example, *Cry1* is regulated by not only E-box but also RRE, and *Per2* is regulated by E-box and other cis-elements such as D-box^3,6,7^. The interlocked oscillatory model is based on studies of behavioral rhythms of mutant mice deficient for core clock genes. *Bmal1* knockout mice^8^ and double knockout mice of *Clock* and its paralog *Npas2*^9^ lost the circadian rhythmicity under constant dark condition. In addition, double deficiency of *Cry1/Cry2*^10^*, Per1/Per2*^11,12^, or *Rev-erbα/Rev-erbβ*^13^ also caused severe attenuation of the behavioral rhythmicity. Deficiency of these transcription factors abolishes transcriptional regulation through E-box or RRE, leading to disruption of the rhythmic transcription of the other clock genes. Because deficiencies of the core clock genes have multiple points of action on the clockwork, it remains unclear how rhythmic transcription of each core clock gene contributes to the circadian clock oscillation and its output.

Recently, mathematical modeling has been employed to substantiate molecular mechanisms underlying various oscillatory systems such as circadian clockwork^14,15^. Although the mammalian circadian clockwork consists of the two interlocking feedback loops, mathematical modeling indicates that a single negative feedback loop with time delay can generate sustained oscillations^16^. Experimentally, synthetic biology using an artificial oscillatory system of microbial consortium revealed autonomous oscillation of transcription by a single negative feedback loop^17^. It should be emphasized, however, that an additional interlocked loop provides robustness of the oscillatory system^17–19^. It is conceivable that the structure consisting of the two interlocking loops is beneficial for the mammalian circadian clock system.

In this study, we focused on a physiological role(s) of the RRE-mediated feedback loop in the circadian clock. In previous studies, functional RREs ([A/T]A[A/T]NT[A/G]GGTCA) were identified in several clock genes, and two closely-positioned RREs are located in the *Bmal1* 5ʹ-UTR region^4,20^. The RRE-mediated transcriptional regulation is expected to determine the abundance of BMAL1 protein, in combination with the protein degradation through posttranslational regulation such as phosphorylation^21–23^. Then, bioluminescence rhythms were detected by using a luciferase reporter including the two RREs^20,24^^.^ While *Bmal1* shows clear mRNA rhythms in most mouse tissues^25,26^, it remains unclear whether the two RREs are required for the expression rhythm of endogenous *Bmal1* gene. Furthermore, it is still elusive to what degree the RRE-mediated rhythmic transcription of *Bmal1* contributes to the circadian clock oscillation. Here, by deleting the highly conserved DNA region including the two RREs, we established mutant cells and mice deficient for the RRE-mediated transcriptional regulation of *Bmal1* gene. We also employed a mathematical model of the mutant, in which we observed constitutive expression of *Bmal1* mRNA. Integration of mathematical modeling and biochemical analysis of the RRE-deficient mutants revealed that the RRE-mediated feedback loop plays a key role in stabilizing the 24-hour timekeeping system through the functional rhythm of BMAL1 protein. The present study provides direct evidence that the coupling of the two feedback loops through RRE and E-box underpins the robust circadian clock oscillation.

## Results

### RRE elements are essential for rhythmic transcription of *Bmal1*

*Bmal1* gene is essential for circadian oscillation^8,27^ (Supplementary Fig. 1), and two RREs in the *Bmal1* 5’-UTR region are highly conserved among mammals (Supplementary Fig. 2a). Luciferase reporters containing the RREs has been widely used for monitoring cellular bioluminescence rhythms^20,24^. Deletion or mutation of these two RRE sequences in a luciferase reporter abolished the bioluminescence rhythm (Supplementary Fig. 2b–e) as was shown previously^20,24,28^, indicating that the two RREs are important for generating rhythms of the reporter. To examine contribution of the two RREs to the transcriptional rhythm of endogenous *Bmal1* gene, we generated mutant NIH3T3 cells and mutant mice that are deleted of the two RREs by using CRISPR-Cas9 system. Specifically, we isolated three independent lines of mutant cells and two lines of mice (named ΔRRE mutants), all of which had a double allelic deletion in the RREs (Fig. 1b and Supplementary Fig. 3). The ΔRRE mutant mice were born in the expected Mendelian ratio with normal morphology. In quantitative RT-PCR analysis, we observed clear *Bmal1* expression rhythm in wild-type (WT) NIH3T3 cells, and the *Bmal1* rhythm was abrogated in the ΔRRE mutant cells (Fig. 1c). Similarly, the *Bmal1* expression rhythm was abolished in the mutant mouse liver, kidney, heart, and epididymal white adipose tissue (eWAT) (Fig. 1c). These results demonstrated that transcriptional regulation through the two RREs located in the *Bmal1* 5ʹ-UTR region is essential for the circadian expression of endogenous *Bmal1* gene.

The amounts of *Bmal1* mRNA in the ΔRRE mutant NIH3T3 cells were maintained at a high level that was comparable to the peak of the *Bmal1* mRNA rhythm in WT, whereas the mutant mouse tissues showed constant expression levels that were in the middle or lower range (Fig. 1c). These data suggest that the balance in transcription factors between the activators (RORs) and the repressors (REV-ERBs) of the RRE-mediated transcription is different among cells and tissue types. This idea is supported by the fact that ROR isoforms are expressed at quite different levels among tissues, whereas REV-ERBs show relatively ubiquitous expression in almost all tissues^25,27^.

### Circadian oscillation persists without transcriptional rhythm of *Bmal1*

**Fig. 2:**
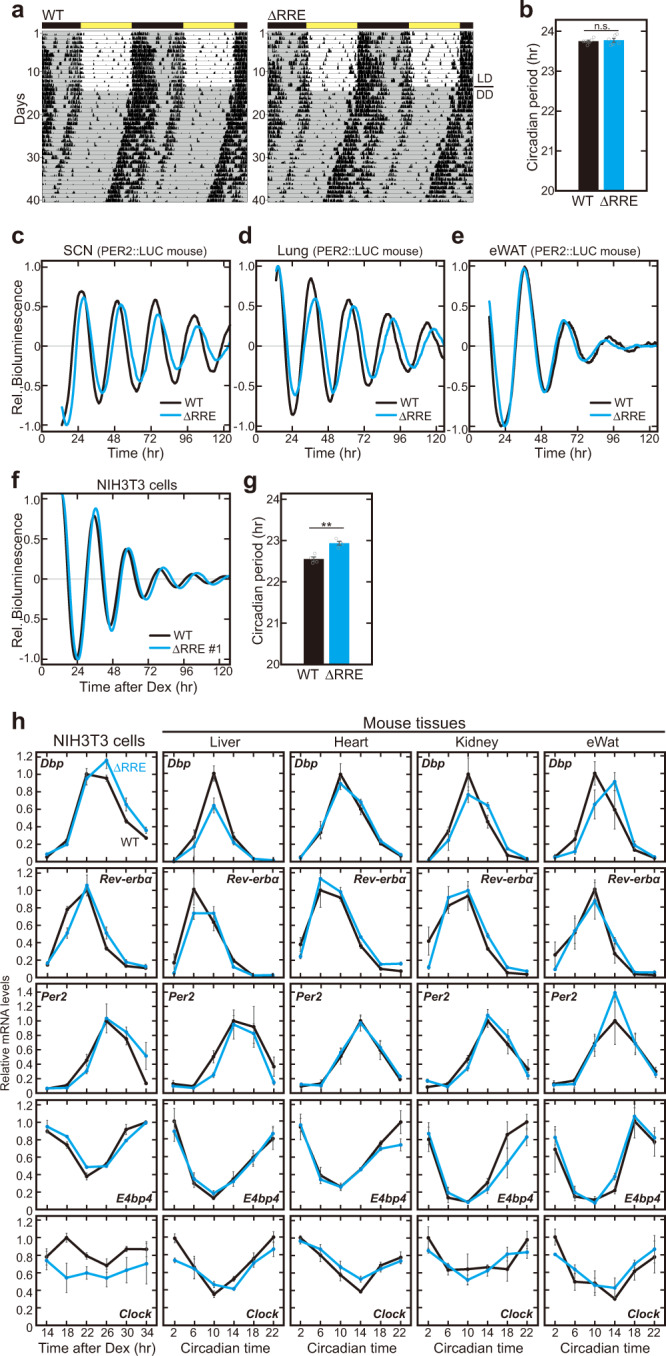
Circadian oscillation is apparently maintained in the absence of the rhythmic transcription of *Bmal1*. **a** Double-plotted wheel-running activities of representative ΔRRE homozygous and littermate WT mice. Horizontal black and yellow bars above each actogram indicate dark and light phases in the light-dark (LD) cycle, respectively. **b** The circadian period of the activity rhythm under the DD condition determined via a chi-square periodogram procedure based on the locomotor activities in days 11 to 24 after the start of DD condition. Data are means ± SEM from 6 WT mice and 6 homozygous ΔRRE mice. Two-sided Student’s *t* test, n.s., *P* ≥ 0.05 *vs*. WT. Source data are provided as a Source Data file. **c**–**e** Representative relative bioluminescence rhythms in the SCN slice (**c**), lung (**d**) and eWat (**e**) of PER2::LUC-WT and PER2::LUC-ΔRRE mice. The tissues are cultured on the Millicell membrane for the long-term monitoring. Blue and black lines indicate the ΔRRE mutants and WT (littermate) controls, respectively. **f**, **g** Representative relative bioluminescence rhythms from the ΔRRE and WT NIH3T3 cells. A Luciferase reporter containing RREs was transiently transfected to monitor the bioluminescent rhythms. Cells were synchronized by 2-hr pulse treatment with dexamethasone just before the monitoring. The circadian periods of the ΔRRE mutant and WT cells are shown in panel (**g**). Data are means ± SEM (*n* = 4). Two-sided Student’s *t* test, **P < 0.01 vs. WT (*P* = 0.0016). Source data are provided as a Source Data file. **h** Temporal mRNA expression profiles of five clock genes (*Dbp, Rev-erbα*, *Per2*, *E4bp4* and *Clock*) in NIH3T3 cells and mouse tissues. The mRNA level of each gene was normalized by *Rps29*. *Bmal1* mRNA levels in the ΔRRE mutant cells and mouse tissues were judged as arrhythmic by the analysis of BIO_CYCLE. Also, *Clock* mRNA level in NIH3T3 cells was judged as arrhythmic in both WT and the ΔRRE mutant. Indicated cells and tissues were harvested at 4-hr intervals, followed by quantitative RT-PCR. Data are means ± SEM (*n* = 3 for WT, *n* = 4 for ΔRRE). Blue and black lines indicate the ΔRRE mutants and WT (littermate) controls, respectively.

To evaluate roles of the *Bmal1* expression rhythm at a behavioral level, we monitored wheel-running rhythms of the ΔRRE mutant mice. The mutant mice exhibited normal daily rhythms entrainable to 12-hr light/12-hr dark (LD) cycles (Fig. 2a and Supplementary Fig. 4). When released to constant dark (DD) conditions, the mutant mice showed stable behavioral rhythms according to their internal circadian clock (Fig. 2a and Supplementary Fig. 4), in contrast with *Bmal1* knockout mice that exhibited complete loss of the rhythmicity in DD^8^. No significant difference was observed in free-running period in DD between the ΔRRE mutant and control mice (Fig. 2b), indicating that the *Bmal1* expression rhythm is not required for the behavioral rhythm. To monitor the circadian oscillation at a tissue level by a bioluminescence reporter system, the *Bmal1*-ΔRRE mutant mice were crossed with PER2::LUC mice. We found that clear bioluminescence rhythms were maintained in slices of suprachiasmatic nucleus (SCN), lung, and eWAT of the double knock-in mice (Fig. 2c–e, Supplementary Fig. 5). Furthermore, in the ΔRRE mutant of NIH3T3 cells, we observed clear circadian rhythm, when the cells were transiently transfected with the luciferase reporter (Fig. 2f, g and Supplementary Fig. 6a, b). To investigate whether other clock genes maintain their expression rhythms, we performed qRT-PCR analysis of the ΔRRE mutant cells and mouse tissues, and found clear mRNA rhythms of both E-box-controlled genes (*Dbp*, *Rev-erbα*, and *Per2*) and RRE-controlled genes (*E4bp4* and *Clock*) (Fig. 2h). We also observed clear circadian rhythms of a series of clock proteins in cell nuclei of the mutant liver, similar to what were observed in WT liver nuclei (Supplementary Fig. 7a, b). Taken together, these results indicate that the RRE-mediated rhythmic transcription of *Bmal1* gene is dispensable for the circadian oscillation in both cells and mice.

### BMAL1 phosphorylation rhythm is maintained even in the absence of its transcriptional rhythm

**Fig. 3:**
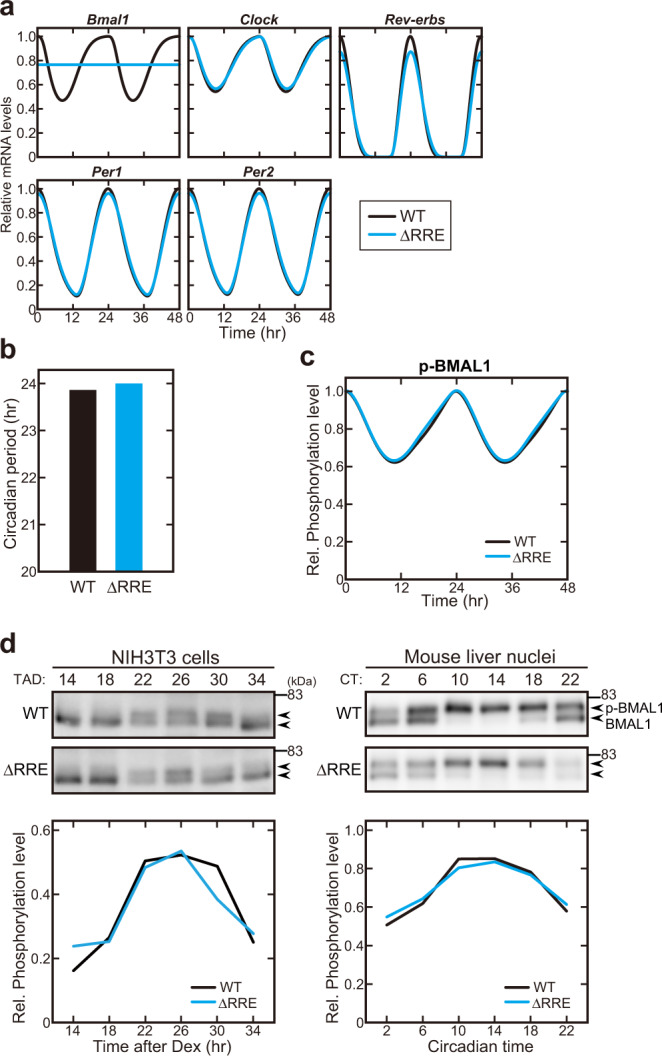
The functional rhythm of BMAL1 is maintained in the absence of the *Bmal1* mRNA rhythm. **a** Simulated temporal profiles of mRNA expression of clock genes. Blue and black lines indicate the ΔRRE mutant and WT models, respectively. **b** Simulated circadian period in the ΔRRE mutant and WT models. **c** Simulated expression profiles of phosphorylated BMAL1 (p-BMAL1) protein in the ΔRRE mutant and WT models. **d** Circadian variation of BMAL1 phosphorylation in the CLOCK-BMAL1 complex in NIH3T3 cells (*Left* panel) and mouse liver nuclei (*Right*). Samples were prepared at 4-hr intervals, followed by SDS-PAGE and immunoblotting using anti-BMAL1 antibody. Quantified data are shown in the *Lower* panels.

To systematically investigate how stable circadian rhythms can be maintained in the absence of the *Bmal1* mRNA rhythm (Fig. 2), we adopted a detailed mathematical model describing the mammalian circadian clock (Kim-Forger model), which accurately captures the change of circadian properties in response to various clock gene mutations and pharmacological inhibitions^14,29–31^. To simulate the circadian clockwork in the ΔRRE mutant, we eliminated the RRE-mediated transcriptional regulation of *Bmal1* gene in the model resulting in the constitutive expression of *Bmal1* mRNA (Fig. 3a) like our experimental data (Fig. 1c). This ΔRRE mutant model generated clear circadian rhythms of other clock genes whose amplitudes were similar to those in the WT model (Fig. 3a). The circadian period of the ΔRRE mutant model was slightly longer than that of the WT model (Fig. 3b) and these simulated results were consistent with the experimental data (Fig. 2c–h).

Even in the absence of the rhythmic *Bmal1* transcription, we found that BMAL1 phosphorylation rhythm was still maintained (Fig. 3c). In particular, the ΔRRE mutant and WT models simulate nearly identical rhythms of the BMAL1 phosphorylation. To examine this prediction, we performed immunoblot analysis of BMAL1 protein phosphorylation, which can be monitored by its band-shift in SDS-PAGE gels^23^. Then we observed time-of-day-dependent band-shifts of the BMAL1 protein bands in the ΔRRE mutant cell lysates and the mutant liver nuclei, with temporal profiles that were quite similar to those observed in WT (Fig. 3d). Taken together, functional rhythm of BMAL1 protein represented by its phosphorylation rhythm^21–23,32^ was maintained even in the absence of the *Bmal1* mRNA rhythm, and this should contribute to the apparently normal rhythmicity of the other clock genes in the ΔRRE mutant (Fig. 2c–h).

### Dual regulation of BMAL1 phosphorylation rhythm leads to circadian oscillation

We investigated the molecular mechanism underlying the phosphorylation dynamics (functional rhythm) of BMAL1 in the ΔRRE mutant by using the mathematical model. We first examined the possibility that rhythmic transcription of *Clock* gene through its RRE^3^ may drive the functional rhythm of BMAL1 in CLOCK-BMAL1 heterodimer, because the heterodimerization is known to regulate the BMAL1 phosphorylation^22,23,33^. In the model, we eliminated the RRE-mediated transcriptional regulation of *Clock* gene in addition to that of *Bmal1* gene. Even under the constant expression of *Clock* and *Bmal1* mRNAs, the BMAL1 phosphorylation rhythm was unaffected (Supplementary Fig. 8a–c), suggesting that the *Clock* expression rhythm plays a minimal role for driving functional rhythm of BMAL1 in the ΔRRE mutant. Experimentally, shRNA-mediated *Clock* knock-down reduced the amplitude of the cellular rhythms in both the ΔRRE mutant and WT cells, and the degree of the amplitude reduction was similar to each other (Supplementary Fig. 8d, e). It was also reported that *Clock* gene is almost constantly expressed in the mouse SCN^20^. Together, these observations support our mathematical model predicting that the expression rhythm of *Clock* gene is dispensable for driving the BMAL1 functional rhythm.

**Fig. 4:**
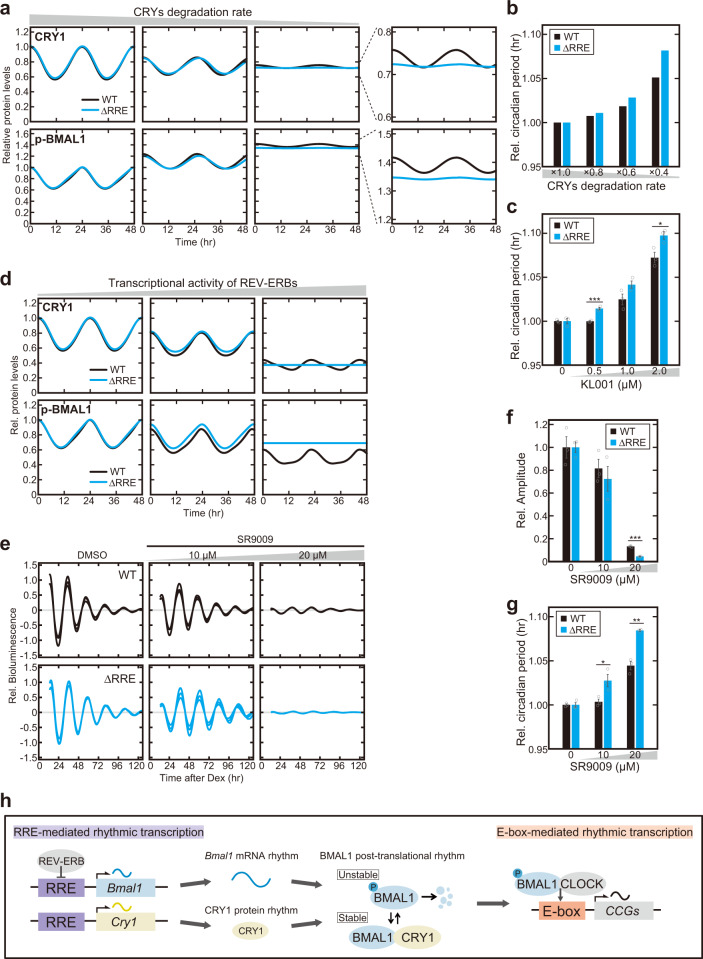
Circadian oscillation in the ΔRRE mutant is sensitive to perturbations of CRY1 protein rhythm. **a** Simulated temporal expression profiles of CRY1 (*Upper* panel) and phosphorylated BMAL1 (*Lower*) depending on CRYs degradation rate. The degradation rate of CRYs in the nucleus was decreased by 60% (Center panel) and 80% (Right). Enlarged figures of the *Right* panel are shown next to them. Blue and black lines indicate the ΔRRE mutant and WT models, respectively. **b** Simulated circadian period change in the ΔRRE mutant and WT models when the CRYs degradation rate was decreased (i.e., CRYs were more stabilized) by 20%, 40% and 60%. In each of the mutant and WT, the circadian period of the original state was normalized to 1.0. **c** Effect of KL001 on the circadian period change of the bioluminescence rhythm in the ΔRRE mutant and WT NIH3T3 cells. Data are means ± SEM (*n* = 3). Two-sided Student’s *t* test, *P < 0.05; ***P < 0.001 vs. WT (0.5 µM, *P* = 0.00062; 2.0 µM, *P* = 0.027). Source data are provided as a Source Data file. **d** Simulated temporal expression profiles of CRY1 (*Upper* panel) and phosphorylated BMAL1 (*Lower*) depending on the repressor activity of REV-ERBs. The REV-ERBs activity increases by decreasing the dissociation constant of REV-ERBs to RREs of clock genes by 50% (Center panel) and 90% (Right). **e**–**g** Effect of SR9009, an agonist of REV-ERBs, on the bioluminescence rhythm in the ΔRRE mutant and WT NIH3T3 cells. Relative amplitudes of the cellular rhythms are shown in (**f**) as means ± SEM (*n* = 3). The circadian periods of the cellular rhythms are shown in (**g**) as means ± SEM (*n* = 3). Two-sided Student’s *t* test, *P < 0.05; **P < 0.01; ***P < 0.001 vs. WT (**f**, 20 µM, *P* = 0.000019; **g**, 10 µM, *P* = 0.035; 20 µM, *P* = 0.0012). Source data are provided as a Source Data file. **h** A model for dual regulation of BMAL1 functional rhythm that leads to the robust circadian oscillation. The RRE-mediated rhythmic transcription of two clock genes *Bmal1* and *Cry1* cooperates to generate the functional rhythm of BMAL1 protein. The rhythmic transcription of *Bmal1* and the rhythmic post-translational regulation of BMAL1 protein via interaction with CRY1 lead to the E-box-mediated rhythmic transcription.

We next focused on CRYs-mediated regulation of the BMAL1 functional rhythm, because CRY proteins are known to suppress the BMAL1 phosphorylation and consequently stabilize BMAL1 protein^21,23^. To investigate whether CRYs may play a key role for the circadian oscillation in the absence of the *Bmal1* mRNA rhythm, we perturbed the rhythmic expression of CRYs in the mathematical models of the ΔRRE mutant and WT. When the degradation rates of CRY1 and CRY2 were decreased (*i.e*., CRYs were more stabilized), the BMAL1 phosphorylation rhythm was attenuated in the WT model and completely abolished in the ΔRRE mutant (Fig. 4a). We found that such disruption of BMAL1 phosphorylation rhythm mainly originates from the perturbation of CRY1 degradation rate via the simulation (Supplementary Fig. 9). Furthermore, the model simulation showed that the perturbation of CRY proteins stability caused a larger change in the circadian period in the ΔRRE mutant than in the WT model (Fig. 4b and Supplementary Fig. 10a). To confirm this prediction, the ΔRRE mutant and WT cells were treated with KL001, which stabilizes CRY proteins by binding to their FAD-binding pockets^34^. Indeed, the circadian period of the bioluminescence rhythm was more lengthened by KL001 in the mutant cells than in the WT cells (Fig. 4c and Supplementary Fig. 10b). The more significant period-lengthening effect in the mutant raises the idea that the CRY protein rhythm and the *Bmal1* mRNA rhythm cooperate to drive the normal circadian oscillation.

This idea led us to investigate whether the ΔRRE mutant and WT differently respond to perturbation of *Cry* transcription. In the mathematical model, we increased the transcriptional activity of REV-ERBs that is known to suppress transcription of *Cry1* gene through its RRE elements^6^. This completely abolished the rhythmicity of CRY1 protein and phosphorylated BMAL1 levels in the ΔRRE mutant model but not in the WT model (Fig. 4d). Experimentally, NIH3T3 cells were treated with SR9009, an agonist of REV-ERBs^35^, and indeed, we observed that the amplitude of the bioluminescence rhythm was more severely reduced in the ΔRRE mutant cells than in the WT cells (Fig. 4e, f). Furthermore, the SR9009 treatment lengthened the circadian period, and the effect was significantly stronger in the ΔRRE mutant cells (Fig. 4g and Supplementary Fig. 10c). These observations demonstrated that the ΔRRE mutation increased susceptibility of the circadian oscillation to perturbations of CRY1 protein.

Taken together, we focused on the BMAL1 phosphorylation rhythm in the absence of the *Bmal1* transcriptional rhythm, and found that the CRY1 protein rhythm is a key contributor to maintaining the apparently normal circadian oscillation in the ΔRRE mutant cells. The RRE-mediated transcriptional rhythms of the two clock genes *Bmal1* and *Cry1* cooperate to generate the functional rhythm of BMAL1 protein and hence leads to the robust circadian rhythm of E-box-mediated transcription (Fig. 4h).

### Physiological roles of the RRE-mediated feedback loop

**Fig. 5:**
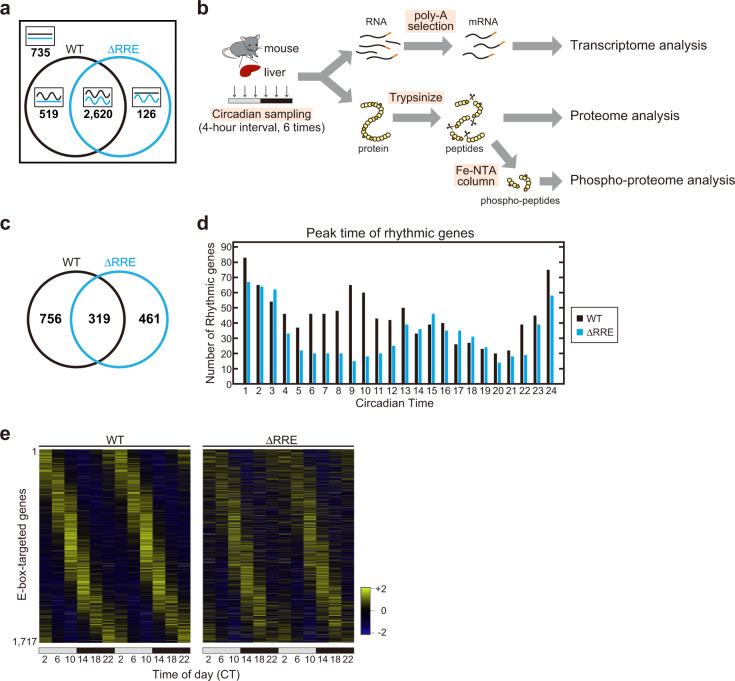
The RRE-mediated feedback loop increases the robustness of the circadian clock. **a** Venn diagram (sizes not to scale) depicting the number of parameter sets that permit oscillations in the ΔRRE mutant and WT models. Four thousand parameter sets were obtained by randomly perturbing the values of the model parameters. **b** The experimental procedures for the transcriptome and proteome analyses. The livers were harvested from the ΔRRE homozygous mice and the control WT mice at 4-hr intervals. The cDNA library was prepared from poly(A)-tailed RNA, and sequenced for transcriptome analysis. The trypsinized peptides were subjected to LC–MS/MS analysis for total proteome analysis, and the phosphorylated peptides were enriched by FeNTA column for phospho-proteome analysis. **c** Venn diagram (sizes not to scale) depicting the number of rhythmic genes in the transcriptome analysis of the ΔRRE mutant and WT mouse liver. **d** A histogram of the number of rhythmic genes peaking at each circadian time. Blue and black indicate the ΔRRE mutants and WT littermates, respectively. **e** Heat maps of mRNA levels of 1717 E-box-targeted genes expressed in the RNA-Seq. Genes were ordered by their peak phases in WT mice from early day to late night. The FPKM values were normalized so that the mean and the variance were set to 0 and 1, respectively, for each row of the maps.

The circadian molecular oscillation in the ΔRRE mutant was apparently normal (Figs. 2, 3) but fragile when perturbations were given to CRY1 protein (Fig. 4). The mRNA levels of *Bmal1* show clear circadian rhythms in most tissues of mice^25^ (Fig. 1c) owing to its upstream RREs, which are highly conserved in mammals^20^ (Supplementary Fig. 2a). These lines of evidence raised the possibility that the RRE-mediated feedback loop increased the robustness of the circadian rhythm of E-box-mediated transcription through the functional rhythm of BMAL1. To compare the robustness against environmental perturbations between the ΔRRE oscillator and WT oscillator, we randomly perturbed the model parameter set by changing the parameters within a range of ±50% (see Materials and Methods for details), and examined whether the perturbed parameter sets permit circadian oscillations in the ΔRRE mutant and WT models. Among randomly perturbed 4,000 model parameter sets, 2,620 parameter sets allowed the both models to oscillate (Fig. 5a), and this result is consistent with the apparently normal oscillation in the ΔRRE mutant cells and mice (Fig. 2). Importantly, we found that 519 parameter sets permitted oscillation only in the WT model, whereas 126 parameter sets permitted oscillation only in the mutant model. The fraction of the parameter sets leading to rhythms was not affected even when the criterion for oscillation was modified to be stricter and the total number of perturbed parameters sets was varied (Supplementary Fig. 11). Note that perturbations of parameters associated with clock proteins including REV-ERBs and CRYs (Supplementary Fig. 12) led to a larger decrease of the rhythm amplitude in the ΔRRE model than WT model. Because REV-ERBs regulate the rhythmicity of *Cry1* transcription through the intronic RRE, this result indicates the importance of the dual regulation of BMAL1 functional rhythms, which is consistent with our experimental data (Fig. 4). These results demonstrate that the RRE-mediated feedback loop plays a role in stabilizing the circadian oscillation in the face of external perturbations.

To further explore the advantage of the interlocking structure composed of the two feedback loops, we examined the effects of the ΔRRE mutation on global changes of transcriptome (Supplementary Fig. 13, Supplementary Data 1), proteome (Supplementary Fig. 14, Supplementary Data 2, 3) and phospho-proteome (Supplementary Fig. 15, Supplementary Data 4, 5). We collected livers from the ΔRRE mutant and WT mice at 6 time points (4-hr intervals) throughout the day (Fig. 5b). In the liver transcriptome analysis, we found that the number of rhythmic genes was reduced in the mutant (Fig. 5c). Such reduction was observed particularly in a population of genes peaked at CT6-CT12 (Fig. 5d, Supplementary Fig. 13). Because the circadian expressions of E-box-regulated genes are known to peak at around CT6-CT12 in the mouse liver^3^, we next compared the rhythmicities of the E-box-regulated genes between the ΔRRE and WT livers. Among the E-box-targeted 2186 genes identified in our previous CLOCK-ChIP-Seq study^36^, 1717 genes were detected as expressed in the present RNA-seq analysis. The heat map of the 1,717 genes showed that the temporal expression profiles of the E-box-targeted genes were markedly affected by the ΔRRE mutation (Fig. 5e). The circadian expressions of the rhythmic genes peaked at CT6-CT12 (including many E-box-regulated genes) might be perturbed by the impaired E-box-mediated transcriptional rhythms in the ΔRRE mutants. This observation raised the possibility that the RRE-mediated feedback loop through the *Bmal1* rhythm affected the circadian rhythm of E-box-mediated transcription through the functional rhythm of BMAL1. In the simulation, the ΔRRE mutation caused a lengthened phase relationship between peak times of phosphorylated BMAL1 rhythm and mRNA rhythm of *Rev-erbα*, an E-box-regulated gene (Supplementary Fig. 16). This altered relationship would be due to the slower decrease in abundance of active BMAL1 protein (not sequestered by the repressors) in the ΔRRE mutant model. Consequently, the E-box-mediated transactivation driven by active BMAL1 may be delayed in the ΔRRE mutant, leading to lengthening of the circadian period observed in the cellular bioluminescence rhythm (Fig. 2f, g and Supplementary Fig. 6a, b) and the mathematical model (Fig. 3b).

Collectively, the mathematical modeling and experiments on the ΔRRE mutant demonstrated that the RRE-mediated feedback loop plays pivotal roles in stabilizing the circadian timekeeping system and organizing its appropriate output. The present study suggests that the structure consisting of the two interlocking feedback loops provide evolutionary benefit to the circadian clocks.

## Discussion

Transcriptional feedback regulations through DNA *cis*-elements play important roles in various physiological functions. In the circadian clockwork of various organisms, a core negative feedback loop and an additional feedback loop(s) together generate stable oscillations^1^. In the present study, we provide evidence for the physiological roles of the RRE-mediated additional feedback loop in the mammalian circadian clock. We generated the ΔRRE mutant cells and mice that were deficient for circadian expression of endogenous *Bmal1* gene (Fig. 1), and we found that the apparently normal circadian rhythms were maintained in the absence of the RRE-mediated feedback loop (Figs. 2, 3). Integration of mathematical modeling and biochemical analysis of the RRE-deficient mutants, however, revealed that the RRE-mediated feedback loop plays a key role as one of the twin pillars that stabilize the 24-hour timekeeping system (Fig. 4). The two of the RRE-mediated *Bmal1* transcriptional rhythm and CRY1 protein rhythm coordinately drive the BMAL1 phosphorylation rhythm, leading to the E-box-mediated rhythmic transcription and hence providing the robust circadian clock oscillation against the perturbation (Fig. 5a). In addition to the resilience to perturbations, our transcriptome analysis demonstrated a crucial role of *Bmal1* transcriptional rhythm for driving appropriate temporal expressions of E-box-regulated genes (Fig. 5c–e). The reduction of the E-box-mediated gene expression rhythms appears to be due to the loss of the *Bmal1* transcriptional rhythm, though we do not exclude any posttranscriptional effects of the 41-base deletion in 5’-UTR of *Bmal1* mRNA.

A series of knockout studies in mice have identified the core clock genes that are essential to generate circadian rhythms (e.g., *Bmal1*^8^, *Clock* and its paralog *Npas2*^9^, *Cry1/Cry2*^10^*, Per1/Per2*^11,12^ and *Rev-erbα/Rev-erbβ*^13^). As those clock genes are rhythmically transcribed, a natural follow-up question is the role of their rhythmic expressions. A recent study showed that a mutation of a noncanonical E-box (E’-box) located in the promoter region of *Per2* gene perturbed circadian rhythms in cells and mice^37^. Interestingly though, clear *Per2* mRNA rhythms were maintained in the SCN and liver of the mutant mice^37^, suggesting the presence of other circadian regulation(s) at *Per2* transcript level. In comparison, the present study is the first to generate mutant cells and mice in which the expression rhythms of a core clock gene are completely abolished (Fig. 1c). Although the tamoxifen-induced whole-body deficiency of *Rev-erbα* and *Rev-erbβ* genes in mice severely attenuated the circadian rhythmicity of the wheel-running activity^13^, it was recently reported that SCN-specific *Rev-erbα/β*-deficient mice showed a clear behavioral rhythm with a shorter circadian period^38^. This finding is consistent with the previous studies that *Bmal1* expression driven by a non-circadian promoter was able to rescue circadian clock oscillation in *Bmal1*-KO mice^39^ and cells^27^. In the present study, we manipulated the rhythmic transcriptional regulation of endogenous *Bmal1* gene, and found that BMAL1 functional rhythm still persisted due to the post-translation regulation of BMAL1. This result suggests that functional rhythms of the core clock proteins are generated by not only rhythmic transcription but also other rhythmic regulations such as post-translational modifications. Our finding illustrates the risk of assuming that the constitutive expression of clock genes completely abolishes the functional rhythmicities of their encoded proteins.

Here, we discuss the molecular mechanism of how the perturbations of CRY1 protein more strongly affected the circadian oscillation of the ΔRRE mutant than that of WT (Fig. 4). Taking consideration of previous studies that CRY1 suppresses phosphorylation of BMAL1 and stabilizes it^21,23^, we conclude that the RRE-mediated rhythmic transcription of *Cry1* regulates the post-translational rhythm of BMAL1 protein even in the absence of transcriptional rhythm of *Bmal1*. The circadian period and amplitude of the ΔRRE mutant were more susceptible to the alteration of CRY1 protein levels, suggesting that the BMAL1 functional rhythm was regulated by the two pathways; the *Bmal1* transcription rhythm and the CRY1 protein rhythm (Fig. 4h). Note that another possible explanation for the results (Fig. 4) is not excluded. We previously showed that proper stoichiometric balance between activators (BMAL1/CLOCK) and repressors (PERs/CRYs) of E-box-mediated transcription was key to the sustained oscillation^14^. Our mathematical model predicted that an additional negative feedback loop improves the stoichiometric balance^14^, which was then supported by a synthetic oscillator^17^. The loss of the RRE-mediated feedback loop can reduce the stability of the stoichiometric balance between the activators and repressors of E-box. Thus, it is possible that the oscillation of the ΔRRE mutant is fragile to perturbations of not only CRY protein but also various core clock components. Indeed, when the parameters in our mathematical model were randomly perturbed, the ΔRRE mutant model is more likely to lose rhythms compared to the WT model (Fig. 5a).

For most organisms, maintaining the period of the circadian clock close to 24 hour is advantageous for survival^40,41^. It has been reported that some genetic variations which shorten or lengthen the circadian period cause physiological disorders due to desynchronization between the environmental 24-hour cycle and intrinsic rhythms^42,43^. In the ΔRRE mutant cells and mouse tissues, the expression rhythms of some clock genes were slightly phase-delayed when compared with WT (Fig. 2h and Supplementary Fig. 7). These apparent phase-delays could be due to the lengthened circadian period of the peripheral clock in the mutant. Consistently, the period of the bioluminescence rhythm was significantly lengthened by the ΔRRE mutation in NIH3T3 cells (Fig. 2f, g and Supplementary Fig. 6a, b). Thus, the circadian period in the peripheral tissues became longer by the mutation, but no significant difference was detected in the circadian period of the SCN-driven rhythms in behavioral activity between the mutant and WT mice (Fig. 2a, b), suggesting that the central and peripheral clocks in the mutant are locked in phases different from those in WT. The delayed phase of biological functions in the peripheral tissues of the mutant mice could lead to some physiological disorders. In our GO analysis of the liver proteome data, proteins upregulated by the ΔRRE mutation were enriched in biological pathways including “immune system process”, “response to stress” and “plasminogen activation” (Supplementary Fig. 14a and Supplementary Data 3). On the other hand, proteins downregulated by the mutation were enriched in such pathways as “small molecule metabolic process”, “cellular amide metabolic process” and “mitochondrial translation” (Supplementary Fig. 14b and Supplementary Data 3). The deficiency of the RRE-mediated rhythmic transcription of *Bmal1* might result in a pathological condition similar to chronic inflammation and metabolic disorders, possibly due to the phase-delayed profile of the gene expression. Molecularly, Kinase-Substrate Enrichment Analysis (KSEA) based on kinase-substrate relationships^44^ revealed that GSK3β was upregulated but AKT1 and PKC were downregulated in the mutant liver (Supplementary Fig. 15b,c and Supplementary Data 5). These changes of the kinase signaling activities due to the mutation might result in physiological alterations reflected in the GO analysis.

Collectively, we conclude that the highly conserved RRE sequences in the *Bmal1* gene loci confer perturbations-resistance to the circadian clock system and enable well-organized circadian gene expressions in mouse peripheral tissues, which may prevent pathological disorders.

## Methods

### Cell culture

NIH3T3 cells (RIKEN Cell Bank) were maintained at 37 °C under 5% CO_2_, 95% air in Dulbecco’s modified Eagle’s medium (DMEM) (Sigma) supplemented with 10% fetal bovine serum (FBS) and Pen Strep (Gibco) containing final 100 units/mL penicillin and final 100 µg/mL streptomycin.

### Genome editing for generating mutant cell lines

A pX330 vector containing SpCas9 and a chimeric guide RNA (Addgene, cat. no. 42230)^45,46^ was digested by BbsI. For deleting the RRE elements located in the *Bmal1* 5’-UTR region, two sgRNA sequences (Table S1) were designed using an online software, CRISPR direct^47^ (Supplementary Fig. 3). The oligonucleotides containing the sgRNA sequences were synthesized (Sigma), phosphorylated, annealed, and ligated with the pX330 vector. NIH3T3 cells were transfected with the plasmids by using Lipofectamine3000 Reagent (Thermo Fisher scientific). After the transfection, cells were seeded at a density of one cell per well in 96 well plates. Wells containing a single colony derived from a single cell were selected. The genome DNA sequences of the target site were checked by direct Sanger sequencing.

### Real-time monitoring of rhythmic gene expression

We performed real-time monitoring of luciferase expression as described previously^48^ with minor modifications. Briefly, NIH3T3 cells plated on 35-mm dishes were transiently transfected with *Bmal1*-luc/pGL4.12. After 24 hrs, the cells were treated with 0.1 µM (final) dexamethasone (Sigma) for 2 hrs to synchronize circadian phase of the cellular clocks in the dish. Then the media were replaced by recording media: phenol red-free DMEM (Sigma) supplemented with 10% FBS, 3.5 g/L glucose, Pen Strep (Gibco) containing 100 units/mL penicillin and 100 µg/mL streptomycin, 0.1 mM luciferin, and 10 mM 4-(2-hydroxyethyl)-1-piperazineethanesulfonic acid (Hepes)-NaOH (pH 7.0) at the final concentrations. The time point of this medium change was defined as Time After Dex (TAD) 0. The bioluminescence signals were continuously recorded for 6 to 8 days in air with Dish Type Luminescencer, Kronos Dio (ATTO) or LumiCycle (Actimetrics) at 37 °C, unless otherwise mentioned.

For knockdown of *Clock*, NIH3T3 cells were transiently transfected with 1 ng or 3 ng of *Clock* shRNA in combination with *Bmal1*-luc/pGL4.12. Target sequence 5’-GAACA TCAGG CTATG ATTAC T-3’ was inserted into pSilencer 3.1-H1 puro vector (Thermo Fisher scientific), and its empty vector was used as a negative control.

To perturb CRYs protein rhythms, SR9009 (Cayman Chemical Company) or KL001 (Cayman Chemical Company) was dissolved in DMSO and mixed with the recording media. For the control, the same volume of DMSO was added as vehicle to the recording media.

Raw data of the bioluminescence rhythms were smoothed by 2-hr moving averages and were detrended by subtracting 24-hr centered moving averages. The highest and lowest levels of bioluminescence in each cycle were defined as the peak and trough, respectively. The circadian periods were calculated by using three peaks and three troughs in each dish. For calculating the amplitudes of the rhythms, the areas under the 4 curves (from the 1st trough to the 2nd peak) of the detrended data were divided by the length of time in this range.

### RNA extraction from cells

NIH3T3 cells plated on 35-mm dishes were treated with 0.1 µM (final) dexamethasone (Sigma), and after 2 hrs, the media were replaced with a fresh one. The cell lysates were collected every 4 hr from 14 hr after the medium change and lysed with TRIzol reagent (Invitrogen). Total RNAs were prepared with the RNeasy Mini Kit (Qiagen) according to the manufacturer’s protocol.

### Mice

Mice (C57BL/6 background) were handled in accordance with the Guidelines for the Care and Use of Laboratory Animals at The University of Tokyo. Mice were housed in cages with free access to commercial chow (CLEA Japan) and tap water. The animals were maintained in a light-tight chamber at a constant temperature (23 ± 1 °C) and humidity (55 ± 10%). Unless otherwise mentioned, the ΔRRE mutant mice #1 were used for the experiments in this study.

### Generation of mutant mice

Cas9 protein (Guide-it recombinant Cas9 protein, TaKaRa) and sgRNA (Fasmac) were delivered by electroporation to C57BL/6 J embryos at the pronuclear stage as described previously^49^ with slight modifications. Briefly, the embryos were washed three times with Opti-MEM I (Thermo Fisher Scientific) supplemented with 0.1% polyvinylalcohol (PVA) and once with 0.1% PVA-Opti MEM I containing Cas9 protein (100 ng/µL) and sgRNAs (50 ng/µL each). Then the embryos were placed in a line in the gap of an electrode (LF501PT1-10, BEX, Tokyo, Japan) filled with 0.1% PVA-Opti-MEM I (total 5 µL in volume) containing Cas9 protein and sgRNAs, and electroporation was performed using a CUY21EDIT II electroporator (BEX). After electroporation, survived embryos were cultured in modified Whitten’s medium for a night and were transferred into oviducts of 0.5-day-post-coitum recipients.

### Wheel running behavior analysis

Male mice (5–22 wk old) were individually housed in cages each equipped with a running wheel and were entrained to the 12-hr light/12-hr dark (LD) cycles. Wheel revolutions were recorded under the LD cycles for two weeks and subsequently DD condition for 24 days or longer. Their spontaneous locomotor activities were recorded as the number of the wheel revolutions in 5-min bins and were analyzed with ClockLab software (Actimetrics). The circadian period of the activity rhythm under the DD condition was determined via a chi-square periodogram of the wheel running activities from animals that showed rhythmicity with *P* < 0.001, based on the activity in days 11–24 after the start of DD condition.

### RNA extraction from mouse tissues

Tissues from 8- to 24-wk-old male mice were collected every 4 hr from 26 hrs after the beginning of the DD condition (expressed as projected CT). The livers, kidneys, hearts, and eWATs with 3–4 biological replicates were placed in Lysing Matrix D tubes (MP Biomedicals) with TRIzol reagent (Invitrogen), and homogenized by using Fast-Prep 24 instrument (MP Biomedicals). Total RNAs were prepared with the RNeasy Mini Kit (Qiagen) according to the manufacturer’s protocol.

### Quantitative RT-PCR analysis

For quantification of gene expression, RNAs prepared from NIH3T3 cells and the mouse tissues were reverse transcribed by Go Script Reverse Transcriptase (Promega) with both anchored (deoxythymine) 15 primer and random oligo primers. For quantification of mRNA levels, the cDNAs were subjected to StepOnePlus Real-Time PCR Systems (Applied Biosystems) by using GoTaq Master Mix (Promega) with specific primers of target genes (Table S2). The mRNA levels of clock genes were normalized by that of *Rps29*. The maximum value of WT was normalized to 1.0.

### Real-time monitoring of luciferase expression in mouse tissues

Bioluminescence signals derived from luciferase expression in the SCN, lung and eWAT cultures were recorded as described previously^50^ with some modifications. The *Bmal1*-ΔRRE mice were bred with PER2::LUC knockin mice^51^. The coronal SCN slices (200 µm thick) at the middle of the rostro-caudal axis were prepared from the PER2::LUC-WT and the PER2::LUC-ΔRRE mice by using a vibrating blade tissue slicer (Neo LinearSlicer MT, Dosaka EM). The lung and eWAT samples were prepared by using a razor blade. The tissues were cultured on membranes (Millicell-CM, Millipore) in 35-mm dishes with the recording media. The bioluminescence signals were continuously recorded for 6 to 8 days at 37 °C in air with LumiCycle (Actimetrics).

### Preparation of nuclear fractions of mice liver

Nuclear proteins were isolated as previously described^23^. Briefly, the mouse liver (1 g wet weight) was washed with ice-cold phosphate-buffered saline and homogenized on ice with 9 mL of ice-cold buffer A (10 mM Hepes-NaOH [pH 7.8], 10 mM KCl, 0.1 mM EDTA, 1 mM dithiothreitol [DTT], 1 mM phenylmethylsulfonyl fluoride [PMSF], 4 µg/mL aprotinin, 4 µg/mL leupeptin, 50 mM NaF, and 1 mM Na_3_VO_4_). The homogenate was centrifuged (700 ×g for 5 min), and the resultant precipitate was rinsed twice. The precipitate was resuspended in 2 mL of ice-cold buffer C (20 mM Hepes-NaOH [pH 7.8], 400 mM NaCl, 1 mM EDTA, 5 mM MgCl_2_, 2% [vol/vol] glycerol, 1 mM DTT, 1 mM PMSF, 4 µg/mL aprotinin, 4 µg/mL leupeptin, 50 mM NaF, and 1 mM Na_3_VO_4_). After being gently mixed at 4 °C for 1 hr, the suspension was centrifuged (18,000 × g for 30 min), and the supernatant was used as the nuclear fraction.

### Immunoprecipitation

Immunoprecipitation was performed as previously described^23^ with some modifications. The nuclear fraction of the mouse liver was diluted with two volumes of buffer D1 (20 mM Hepes-NaOH [pH 7.8], 5.5 mM NaCl, 1 mM EDTA, 6.5% [vol/vol] glycerol, 1.5% [vol/vol] Triton X-100, 1 mM DTT, 1 mM PMSF, 4 µg/mL aprotinin, 4 µg/mL leupeptin, 50 mM NaF, and 1 mM Na_3_VO_4_). Both the lysates of NIH3T3 cells and diluted nuclear fractions were incubated at 4 °C for 2 hrs with anti-CLOCK antibody^23^ (CLNT1; D334-3; Medical & Biochemical Laboratories). Protein G-Sepharose 4 Fast Flow (Amersham Biosciences) was then added to this mixture, and mixed gently at 4 °C for 1 hr. The beads were collected by centrifugation for 2 min at 360 × g as the immunoprecipitants.

### Antibodies and immunoblot analysis

Proteins separated by sodium dodecyl sulfate-polyacrylamide gel electrophoresis (SDS/PAGE) were transferred to polyvinylidene difluoride membrane (Millipore). The blots were incubated in a blocking solution: 1% [wt/vol] skim milk in TBS (50 mM Tris-HCl, 140 mM NaCl, 1 mM MgCl_2_ [pH 7.4]) for 1 hr at 37 °C and then incubated overnight at 4 °C with a primary antibody diluted 1:1,000 in the blocking solution. The signals were visualized by an enhanced chemiluminescence detection system (PerkinElmer Life Sciences). The blot membrane was subjected to densitometric scanning, and the band intensities were quantified by using ImageQuant TL (GE Healthcare). To evaluate the phosphorylation rhythm of BMAL1 protein, we calculated the intensity of the up-shifted band (i.e., phospho-BMAL1) relative to the total band intensity at each time point. Uncropped and unprocessed scans of all the blots were provided in Supplementary Figs. 17, 18. The primary antibodies used in this study were as follows: anti-CLOCK^23^ (CLSP3; D333-3; Medical & Biological Laboratories), anti-ARNTL^23^ (B1BH2; D335-3; Medical & Biological Laboratories), anti-PER1 (PM091; Medical & Biological Laboratories), anti-PER2 (PM083; Medical & Biological Laboratories), anti-CRY1 (PM081; Medical & Biological Laboratories), anti-CRY2 (PM082; Medical & Biological Laboratories), anti-DBP (PM079; Medical & Biological Laboratories), anti-NR1D1 (PM092; Medical & Biological Laboratories), and anti-ATF2 (C-19; sc-187; Santa Cruz Biotechnology).

### Preparation of peptides for LC-MS/MS analysis

Enzymatic digestion of mouse liver proteins was performed as previously described^52^ with some modifications. The livers from 9- to 24-wk-old male mice were collected every 4 hr from 26 hrs after the beginning of the DD condition (expressed as projected CT) and the liver blocks frozen in liquid nitrogen were lysed with ice-cold PTS buffer (12 mM sodium deoxycholate [DOC], 12 mM sodium N-lauroylsarcosinate [SLS], 100 mM NH_4_HCO_3_) containing 1% [vol/vol] Phosphatase Inhibitor Cocktail 2 and 3 (Sigma) followed by extensive sonication in an ultrasonic disruptor (UR-21P; TOMY; output power 5 for 2 min). After centrifugation at 18,000 ×g, protein concentrations in the supernatants were quantified by Pierce 660 nm Protein Assay Reagent, and the supernatants were diluted into 2.5 mg/mL proteins with the PTS buffer. Proteins (2.5 mg/mL, 100 µL) in the solution were reduced with final 10 mM DTT at 60 °C for 30 min, and then alkylated by incubation with final 22 mM iodoacetamide [IAA] at 37 °C for 30 min in the dark. The resultant protein sample was diluted with 100 mM NH_4_HCO_3_ solution up to 1 mL and digested with trypsin (Sigma) at 1:100 [wt/wt] by incubation at 37 °C for 18 hrs in the dark. After the digestion, an equal volume of ethyl acetate was added to the sample, and the mixture was acidified with final 0.5% [vol/vol] TFA and then well mixed in order to transfer the detergents into the organic phase. After the sample was centrifuged at 18,000 × g for 1 min at room temperature, the aqueous phase containing peptides was collected. The sample was concentrated by a centrifugal evaporator (EYELA) and desalted using a MonoSpin C18 column (GL Sciences). After 10% of the eluate was dried by the evaporator, it was analyzed by LC-MS/MS as a sample labeled “total peptides.” For the phospho-proteomics, the remaining eluate was dried and applied to High-select Fe-NTA phosphopeptide enrichment kit (Thermo Fisher Scientific). The enriched sample, labeled “phospho-peptides,” was dried and subjected for LC-MS/MS analysis.

### LC–MS/MS-based proteome analysis

LC-MS/MS-based proteome analysis was performed as previously described^52^ with some modifications. The dried and desalted peptides were dissolved in distilled water containing 2% acetonitrile and 0.1% TFA. The LC-MS/MS analyses were performed using a mass spectrometer (Q Exactive Plus, Thermo Fisher Scientific) equipped with a nano ultra-HPLC system (Dionex Ultimate 3000; Thermo Fisher Scientific). The peptides were loaded to the LC–MS/MS system with a trap column (0.3 × 5 mm L-column ODS; Chemicals Evaluation and Research Institute) and a capillary column (0.1 × 150 mm L-column ODS; Chemicals Evaluation and Research Institute) at a flow late of 20 µL/min. The loaded peptides were separated by a gradient using mobile phases A (1% formic acid in distilled water) and B (1% formic acid in acetonitrile) at a flow late of 300 nL/min (0% B for 5 min, 0–30% B for 150 min, 30–50% B for 10 min, 50–95% B for 0.1 min, 95% B for 9.8 min, 95–0% B for 0.1 min, and 0% B for 5 min). The eluted peptides were electrosprayed (2.0 kV) and introduced into the MS equipment (positive ion mode, data-dependent MS/MS). Each of the most intense precursor ions (up to the top 10) was isolated and fragmented by higher collision energy dissociation (HCD) with the normalized collision energy (27%). For full MS scans, the scan range was set to 350–1500 m/z at a resolution of 70,000, and the automatic gain control (AGC) target was set to 3e6 with a maximum injection time of 60 ms. For MS/MS scans, the precursor isolation window was set to 1.6 m/z at a resolution of 17,500, and the AGC target was set to 5e5 with a maximum injection time of 100 ms. The Orbitrap mass analyzer was operated with the “lock mass” option to perform shotgun detection with high accuracy. The raw spectra were extracted using Proteome Discoverer 2.2 (Thermo Fisher Scientific) and searched against the mouse SwissProt database (TaxID 10,090 and subtaxonomies, v2017-10-25) with following settings. The parameter of the cleavage was set to trypsin, and the missed cleavage was allowed up to 2. The mass tolerances were set to 10 ppm for the precursor ion and 0.02 Da for the fragment ion. As for protein modifications, we set carbamidomethylation (+57.021 Da) at Cys as static (fixed) modifications for peptide, oxidation (+15.995 Da) at Met and phosphorylation (+79.966 Da) at Ser and Thr as dynamic (non-fixed) modifications for peptide, and acetylation (+42.011 Da) at amino-terminus as a dynamic modification for protein terminus. The amount of each peptide was semi-quantified using the peak area with Precursor Ions Quantifier in Proteome Discoverer 2.2. According to the manufacturer’s procedure, relative levels of proteins were quantified based on unique peptides without shared peptides. Comparison of the phosphorylated amino acids between the present study and a previous study^53^ revealed similar proportions of phosphorylated residues, i.e., 85.2% phospho-Serine (pS) and 14.8% phospho-Threonine (pT).

### Gene ontology (GO) analysis

GO analysis was performed with open source software ShinyGO v0.61^54^ with the following settings: search species, mouse; *P* value cutoff (FDR), 0.05; gene sets, GO Biological Process. A hierarchical clustering tree was obtained from the top 20 significant terms.

### Kinase-substrate enrichment analysis (KSEA)

KSEA analysis was performed as previously described^44^ with some modifications. The resources for kinase-substrate relationship dataset used in this analysis were PhosphoSitePlus (ver. 6.5.9.3) (https://www.phosphosite.org/homeAction), RegPhos2.0 (http://140.138.144.141/~RegPhos/index.php) and the Universal Protein Resource (Uniprot) (https://www.uniprot.org). For quantitative analysis, we used all the phosphorylation sites that were identified in our phospho-proteome analysis on the mouse livers. The amount of each substrate peptide was normalized by an average of the amounts of the substrate peptide in WT samples, and the normalized values were represented as log2 fold changes (positive and negative values indicate an increase and decrease in abundance of the phosphorylated peptide, respectively). These values were subjected to a Python package, Kinact (ver. 0.3)^55^, in order to estimate protein kinase activities by using the kinase-substrate relationship dataset. A kinase activity score was defined as the average of the log2 fold changes of substrate peptides.

### Circadian rhythm detection

To analyze the circadian rhythmicity, datasets were run through BIO_CYCLE (ver0.9.3)^56^ obtained from CircadiOmics web portal. The range of the circadian period was set to 20-28 hr according to the default settings. Unless otherwise mentioned, rhythmic genes were clarified with three criteria: (1) BIO_CYCLE P-value is less than 0.3, (2) When the expression levels are averaged at each time, the highest expression level (Peak) is 1.5 times higher than the lowest expression level (Trough), and (3) The largest SD value among each time point (SD_MAX_) is within 1.5 times the deference between the Peak and Trough (Amplitude).

### RNA-seq analysis

RNA-Seq analysis was performed as previously described^26^ with minor modifications. The livers from the mutant and WT male mice harvested at six time points throughout the day (CT2, 6, 10, 14, 18, and 22; *n* = 2) were placed in Lysing Matrix D tubes (MP Biomedicals) with TRIzol reagent (Invitrogen), and homogenized by using Fast-Prep 24 instrument (MP Biomedicals). Total RNAs were prepared with the RNeasy Mini Kit (Qiagen), followed by ethanol precipitation. Poly(A)-tailed RNA was isolated from the total RNA by using NEBNext Poly(A) mRNA Magnetic Isolation Module (NEB, #E7490) and NEBNext Ultra II Directional RNA Library Prep Kit (NEB, #E7760). The library was sequenced on a NovaSeq 6000 (150 bp, pair end).

### Mathematical model description

We used a mathematical model to investigate how the circadian rhythms of clock molecules can be maintained even in the absence of the RREs of *Bmal1* genes. Their rhythms were simulated by using the Kim–Forger model^14^. The Kim-Forger model is a detailed mathematical model of the intracellular mammalian circadian clock, which describes the reactions among clock molecules (e.g., phosphorylation and binding) in the SCN by using ordinary differential equations based on mass action kinetics (181 variables and 75 parameters)^14^. The model accurately captured various phenotypes of circadian mutations (e.g., *Rev-erbα*^−/−^). To simulate the rhythms in the ΔRRE mutant cells, the RRE-mediated transcriptional regulation of *Bma1* is eliminated in the model. That is, we adjusted the model to make *Bmal1* mRNA be expressed constitutively whose level is the same to the averaged *Bmal1* mRNA level in the WT model (Fig. 3a) based on the experimental data (Fig. 1c). Similarly, to simulate the rhythms in the *Bmal1*- and *Clock*-ΔRRE mutant cells in Supplementary Fig. 8a-c, the RRE-mediated transcriptional regulation of both *Bmal1* and *Clock* is eliminated. That is, we adjusted the model to make *Bmal1* mRNA and *Clock* mRNA be expressed constitutively whose levels are the same with the averaged levels of *Bmal1* mRNA and *Clock* mRNA (Supplementary Fig. 8a).

To simulate the inhibition of FBXL3-dependent degradation of CRYs in Fig. 4a and Supplementary Fig. 9, we decreased the degradation rate constant for CRY1 and CRY2, uro and urt, in the nucleus, respectively, as done in the previous work^34^. To simulate the increase in transcriptional activity of REV-ERBs in Fig. 4d, we decreased the unbinding rate constant for REV-ERBs to *Bmal1* RREs and *Cry1*/*Clock* RREs, unbinrevb and unbinrev, respectively, and thus decreased the dissociation constant of REV-ERBs to *Bmal1* RREs and *Cry1*/*Clock* RREs.

In Fig. 5a, we randomly perturbed all the values of the model parameters, which represent the transcription rates, translation rates, degradation rates, unbinding rates, phosphorylation rates, and nuclear localization rates, by 50%. Then, we checked whether the WT model and the ΔRRE mutant model simulate rhythmic expression of clock molecules. Specifically, to check whether their level oscillates, we measured the relative amplitude (i.e., maximum abundance minus minimum abundance divided by the maximum abundance) of a core clock gene, *Per2*. The rhythmicity criteria were set from weak rhythmicity (the relative amplitude >0.1) to strong rhythmicity (the relative amplitude >0.4). Note that as the expressions of clock molecules are tightly interlocked in the model, the result of the analysis changes little even if other clock gene expression profiles are used to calculate the relative amplitude.

### Reporting summary

Further information on research design is available in the Nature Research Reporting Summary linked to this article.

## Supplementary information

Supplementary Information

Reporting Summary

Description of Additional Supplementary Files

Dataset 1

Dataset 2

Dataset 3

Dataset 4

Dataset 5

## Data Availability

The RNA-seq data obtained in this study have been deposited in the Gene Expression Omnibus (GEO) under accession code GSE199061. The proteomics data obtained in this study have been deposited in PRIDE with the dataset identifier PXD035414. Also, the processed RNA-seq data and proteomics data are provided in the Supplementary Data files. Source data are provided with this paper.

## Source data

Source Data

## Code availability

The codes and readme file for simulation of the mathematical models of the ΔRRE mutant and WT are available in Zenodo database under accession code [10.5281/zenodo.6512360]. The codes are implemented by MATHEMATICA 12.0.

## References

[1] Dunlap JC (1999). Molecular bases for circadian clocks. Cell.

[2] Wilsbacher LD, Takahashi JS (1998). Circadian rhythms: molecular basis of the clock. Curr. Opin. Genet. Dev..

[3] Ueda HR (2005). System-level identification of transcriptional circuits underlying mammalian circadian clocks. Nat. Genet..

[4] Preitner N (2002). The orphan nuclear receptor REV-ERBα controls circadian transcription within the positive limb of the mammalian circadian oscillator. Cell.

[5] Sato TK (2004). A functional genomics strategy reveals rora as a component of the mammalian circadian clock. Neuron.

[6] Ukai-Tadenuma M (2011). Delay in feedback repression by cryptochrome 1 is required for circadian clock function. Cell.

[7] Yoshitane, H. et al. Functional D-box sequences reset the circadian clock and drive mRNA rhythms. *Commun. Biol*. **2**, 300 (2019). 10.1038/s42003-019-0522-3 PMC6687812 31428688

[8] Bunger MK (2000). Mop3 is an essential component of the master circadian pacemaker in mammals. Cell.

[9] DeBruyne JP, Weaver DR, Reppert SM (2007). CLOCK and NPAS2 have overlapping roles in the suprachiasmatic circadian clock. Nat. Neurosci..

[10] van der Horst GT (1999). Mammalian Cry1 and Cry2 are essential for maintenance of circadian rhythms. Nature.

[11] Bae K (2001). Differential functions of mPer1, mPer2, and mPer3 in the SCN circadian clock. Neuron.

[12] Zheng B (2001). Nonredundant roles of the mPer1 and mPer2 genes in the mammalian circadian clock. Cell.

[13] Cho H (2012). Regulation of circadian behaviour and metabolism by REV-ERB-α and REV-ERB-β. Nature.

[14] Kim JK, Forger DB (2012). A mechanism for robust circadian timekeeping via stoichiometric balance. Mol. Syst. Biol..

[15] Pett JP, Korenčič A, Wesener F, Kramer A, Herzel H (2016). Feedback loops of the Mammalian circadian clock constitute repressilator. PLoS Comput. Biol..

[16] Novák B, Tyson JJ (2008). Design principles of biochemical oscillators. Nat. Rev. Mol. Cell Biol..

[17] Chen Y, Kim JK, Hirning AJ, Josić K, Bennett MR (2015). SYNTHETIC BIOLOGY. Emergent genetic oscillations in a synthetic microbial consortium. Science.

[18] Kim JK (2019). Long-range temporal coordination of gene expression in synthetic microbial consortia. Nat. Chem. Biol..

[19] Tsai TY-C (2008). Robust, tunable biological oscillations from interlinked positive and negative feedback loops. Science.

[20] Ueda HR (2002). A transcription factor response element for gene expression during circadian night. Nature.

[21] Kondratov RV (2006). Post-translational regulation of circadian transcriptional CLOCK(NPAS2)/BMAL1 complex by CRYPTOCHROMES. Cell Cycle.

[22] Kwon I (2006). BMAL1 shuttling controls transactivation and degradation of the CLOCK/BMAL1 heterodimer. Mol. Cell. Biol..

[23] Yoshitane H (2009). Roles of CLOCK phosphorylation in suppression of E-box-dependent transcription. Mol. Cell. Biol..

[24] Akashi M, Takumi T (2005). The orphan nuclear receptor RORalpha regulates circadian transcription of the mammalian core-clock Bmal1. Nat. Struct. Mol. Biol..

[25] Zhang R, Lahens NF, Ballance HI, Hughes ME, Hogenesch JB (2014). A circadian gene expression atlas in mammals: implications for biology and medicine. Proc. Natl Acad. Sci. USA.

[26] Terajima H (2017). ADARB1 catalyzes circadian A-to-I editing and regulates RNA rhythm. Nat. Genet..

[27] Liu AC (2008). Redundant function of REV-ERBalpha and beta and non-essential role for Bmal1 cycling in transcriptional regulation of intracellular circadian rhythms. PLoS Genet..

[28] Hirota T (2010). Transcriptional repressor TIEG1 regulates Bmal1 gene through GC box and controls circadian clockwork. Genes Cells.

[29] Kim JK (2013). Modeling and validating chronic pharmacological manipulation of circadian rhythms. CPT Pharmacomet. Syst. Pharmacol..

[30] Kim DW (2019). Systems approach reveals photosensitivity and PER2 level as determinants of clock-modulator efficacy. Mol. Syst. Biol..

[31] D’Alessandro M (2015). A tunable artificial circadian clock in clock-defective mice. Nat. Commun..

[32] Dardente H, Fortier EE, Martineau V, Cermakian N (2007). Cryptochromes impair phosphorylation of transcriptional activators in the clock: a general mechanism for circadian repression. Biochem. J..

[33] Kondratov RV (2003). BMAL1-dependent circadian oscillation of nuclear CLOCK: posttranslational events induced by dimerization of transcriptional activators of the mammalian clock system. Genes Dev..

[34] Hirota T (2012). Identification of small molecule activators of cryptochrome. Science.

[35] Solt LA (2012). Regulation of circadian behaviour and metabolism by synthetic REV-ERB agonists. Nature.

[36] Yoshitane, H. et al. CLOCK-controlled polyphonic regulation of circadian rhythms through canonical and noncanonical E-boxes. *Mol. Cell. Biol*. **34**, 1776–87 (2014). 10.1128/MCB.01465-13 PMC4019033 24591654

[37] Doi M (2019). Non-coding cis-element of Period2 is essential for maintaining organismal circadian behaviour and body temperature rhythmicity. Nat. Commun..

[38] Adlanmerini, M. et al. REV-ERB nuclear receptors in the suprachiasmatic nucleus control circadian period and restrict diet-induced obesity. *Sci. Adv*. **7**, eabh2007 (2021). 10.1126/sciadv.abh2007 PMC8550249 34705514

[39] McDearmon EL (2006). Dissecting the functions of the mammalian clock protein BMAL1 by tissue-specific rescue in mice. Science.

[40] Pittendrigh CS, Minis DH (1972). Circadian systems: longevity as a function of circadian resonance in Drosophila melanogaster. Proc. Natl Acad. Sci. USA.

[41] Dodd AN (2005). Plant circadian clocks increase photosynthesis, growth, survival, and competitive advantage. Science.

[42] Hirano A (2016). A Cryptochrome 2 mutation yields advanced sleep phase in humans. Elife.

[43] Patke A (2017). Mutation of the Human Circadian Clock Gene CRY1 in Familial Delayed Sleep Phase Disorder. Cell.

[44] Casado P (2013). Kinase-substrate enrichment analysis provides insights into the heterogeneity of signaling pathway activation in leukemia cells. Sci. Signal..

[45] Ran FA (2013). Genome engineering using the CRISPR-Cas9 system. Nat. Protoc..

[46] Cong L (2013). Multiplex genome engineering using CRISPR/Cas systems. Science.

[47] Naito Y, Hino K, Bono H, Ui-Tei K (2015). CRISPRdirect: software for designing CRISPR/Cas guide RNA with reduced off-target sites. Bioinformatics.

[48] Yoshitane H (2012). JNK regulates the photic response of the mammalian circadian clock. EMBO Rep..

[49] Hashimoto M, Takemoto T (2015). Electroporation enables the efficient mRNA delivery into the mouse zygotes and facilitates CRISPR/Cas9-based genome editing. Sci. Rep..

[50] Terajima H, Yoshitane H, Yoshikawa T, Shigeyoshi Y, Fukada Y (2018). A-to-I RNA editing enzyme ADAR2 regulates light-induced circadian phase-shift. Sci. Rep..

[51] Yoo S-H (2004). PERIOD2::LUCIFERASE real-time reporting of circadian dynamics reveals persistent circadian oscillations in mouse peripheral tissues. Proc. Natl Acad. Sci. USA.

[52] Imamura K (2018). ASK family kinases mediate cellular stress and redox signaling to circadian clock. Proc. Natl Acad. Sci. USA.

[53] Brüning F (2019). Sleep-wake cycles drive daily dynamics of synaptic phosphorylation. Science.

[54] Ge SX, Jung D, Yao R (2020). ShinyGO: a graphical gene-set enrichment tool for animals and plants. Bioinformatics.

[55] Wirbel J, Cutillas P, Saez-Rodriguez J (2018). Phosphoproteomics-based profiling of kinase activities in cancer cells. Methods Mol. Biol..

[56] Agostinelli F, Ceglia N, Shahbaba B, Sassone-Corsi P, Baldi P (2016). What time is it? Deep learning approaches for circadian rhythms. Bioinformatics.

