# Supplementary material for: Rhythmic transcription of Bmal1 stabilizes the circadian timekeeping system in mammals
Source: Nat Commun. 2022 Aug 23;13:4652. doi: 10.1038/s41467-022-32326-9 (PMC9399252; doi:10.1038/s41467-022-32326-9)
Supplement: Supplementary file 1 — Supplementary Information [file 41467_2022_32326_MOESM1_ESM.pdf]

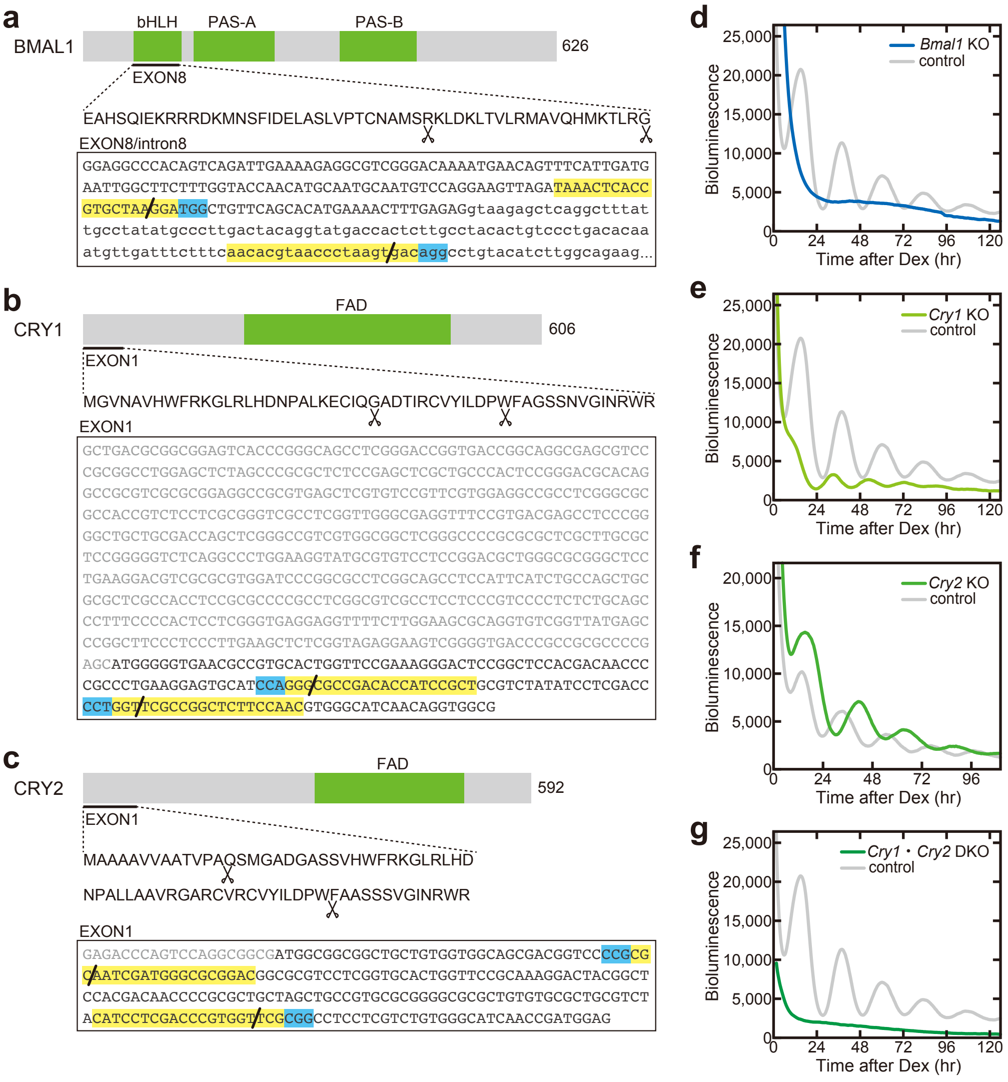

Supplementary Fig. 1.

***Bmal1* KO cells and *Cry1/Cry2* DKO cells lose the circadian clock oscillation.**

(**a-c**) Protein domain structures (*Upper* panels) and edited regions of nucleotide sequences (*Lower*) of *Bmal1* (**a**), *Cry1* (**b**) and *Cry2* (**c**) genes. NIH3T3 cells stably expressing a firefly luciferase reporter under the regulation of a 0.3-kb *Bmal1* promoter were used as previously reported[52]. The NIH3T3 *Bmal1*-luc cells were genome-edited for generating *Bmal1* KO, *Cry1* KO, *Cry2* KO and *Cry1/Cry2* DKO cells. Amino acid sequences to be translated are shown below the protein domain structures. Nucleotide sequences are shown in boxes, where upper case and lower case letters indicate those in exons and introns, respectively. Gray letters indicate the 5'-UTR regions. Cas9-targets and PAM sequences are highlighted in yellow and blue, respectively. The predicted Cas9 cleavage sites are indicated by diagonal lines. (**d-g**) Representative bioluminescence rhythms from the *Bmal1* KO cells (**d**), the *Cry1* KO cells (**e**), the *Cry2* KO cells (**f**) and the *Cry1/Cry2* DKO cells (**g**). These cells were synchronized by 2-hr pulse treatment with dexamethasone just before the bioluminescence monitoring. We thank Dr. Takao Kondo (Nagoya Univ.) for a 24-well dish-type of PMT-Tron and Dr. Hidetoshi Kassai (The Univ. Tokyo) and Dr. Tomoya Shiraki (The Univ. Tokyo; Present address, National Institute of Genetics, Japan) for their helpful discussion about the CRISPR-Cas9 system.

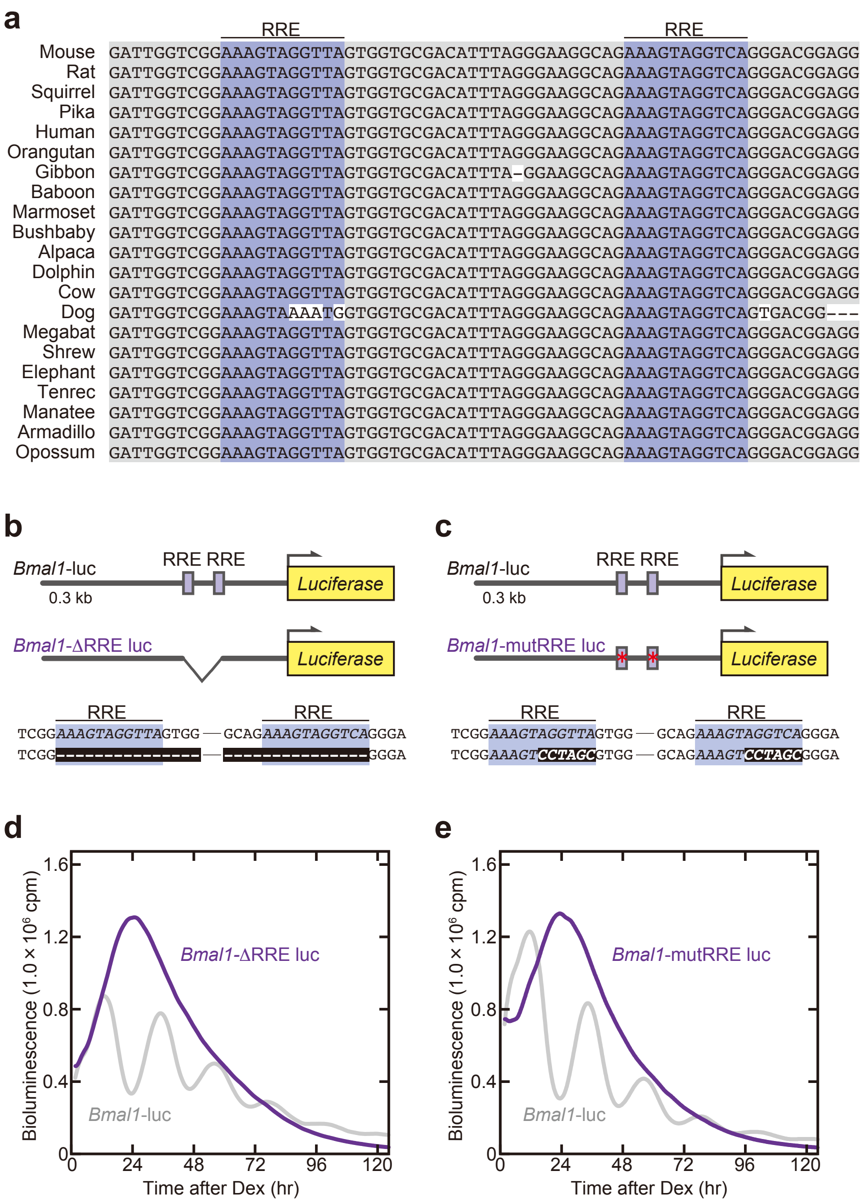

Supplementary Fig. 2.

Two RREs in the 5'-UTR of *Bmal1* are sufficient for gene expression rhythm.

(**a**) Alignment of the nucleotide sequences of mammalian *Bmal1* 5'-UTR containing the two RREs. RRE elements are highlighted in purple and nucleotides diverged from the other sequences are highlighted in white background. The sequence data are obtained from UCSC Genome Browser on Mouse Dec. 2011 (GRCm38/mm10) Assembly. (**b**) Diagrams of the WT *Bmal1*-luc reporter and the *Bmal1*-ΔRRE luc reporter. For generating the *Bmal1*-ΔRRE luc reporter, 47-bp sequence (highlighted in black background) including two RREs was deleted in a *Bmal1*-luc/pGL4.12-hyg plasmid. (**c**) Diagrams of the WT *Bmal1*-luc reporter and the *Bmal1*-mutRRE luc reporter. Plasmids used in this experiment were a *Bmal1*us0.3-luc wild type/pGL3 plasmid and a *Bmal1*us0.3-luc Mut.RORE/pGL3 plasmid, which were reported previously[28]. The mutated sequences were highlighted in black background. (**d-e**) Representative traces of bioluminescence signals recorded from NIH3T3 cells expressing the luciferase reporters. NIH3T3 cells were transiently transfected with either of these plasmids to monitor the bioluminescent rhythm. Data using the mutant luc reporters and the control luc reporters were indicated in purple and gray, respectively.

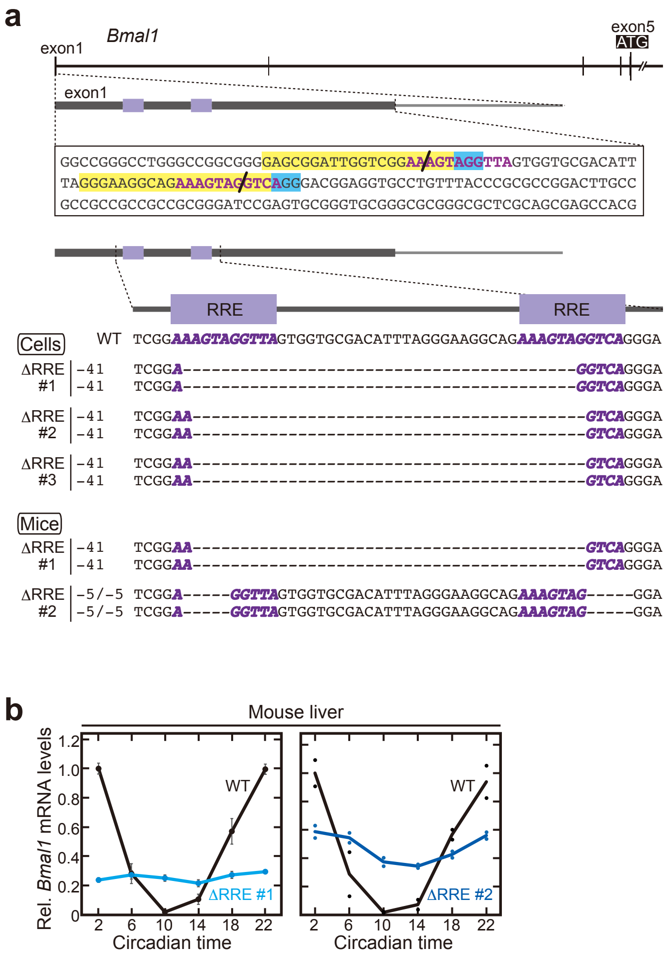

Supplementary Fig. 3.

The mutant cells and mice deficient for two RREs of *Bmal1* gene were generated.

(**a**) The genome structure of the *Bmal1* gene locus (*Upper* panel) and the sequences of ΔRRE cell lines and ΔRRE mouse lines (*Lower*). Thick and thin lines indicate exons and introns of *Bmal1*, respectively. The genome sequence of exon1 is shown in the box. Cas9-targets and PAM sequences are highlighted in yellow and blue, respectively. RRE elements are indicated by two boxes in purple (*Upper*) or bold italic letters in purple (*Lower*). (**b**) Temporal profiles of *Bmal1* mRNA levels in the ΔRRE #1 (n=3 for WT, n=4 for ΔRRE #1, Data are means ± SEM) and ΔRRE #2 (n=2) mice liver. Livers were harvested at 4-hr intervals, followed by quantitative RT-PCR. The mRNA levels of *Bmal1* were normalized by *Rps29*. Blue and black lines indicate the ΔRRE mutants and the controls, respectively. Data of the ΔRRE mutant #1 and its control were reproduced from Fig. 1c.

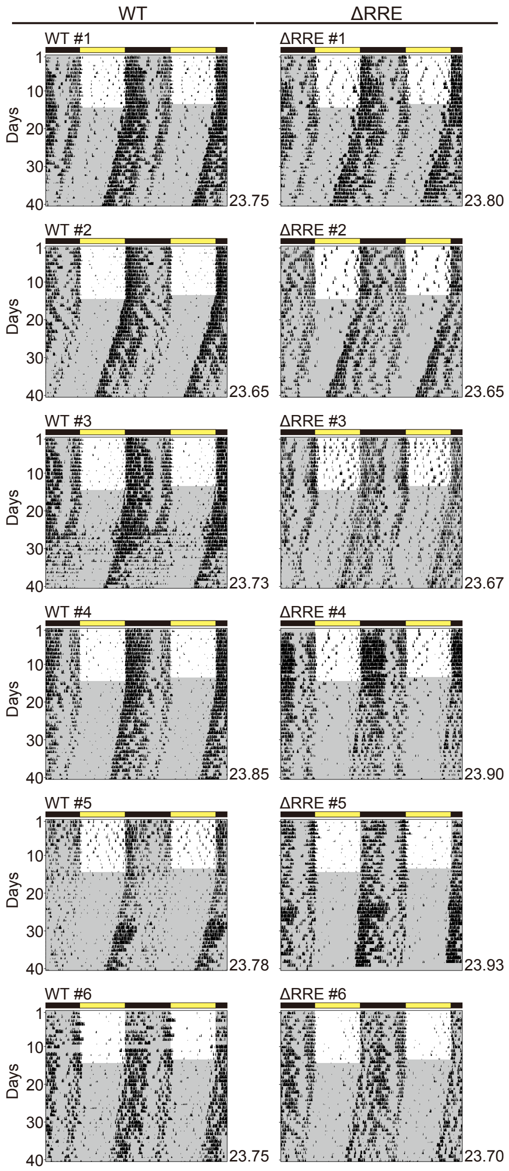

Supplementary Fig. 4.
Wheel-running rhythms of mice were maintained in the absence of the RRE-mediated rhythmic transcription of *Bmal1*.

Double-plotted actograms of wheel-running activities of the ΔRRE homozygous and littermate WT mice. Horizontal black and yellow bars above each actogram indicate dark and light phases in the light-dark (LD) cycle, respectively. The circadian periods were determined via a chi-square periodogram procedure based on the locomotor activities in days 11 to 24 after the start of DD condition. The determined circadian periods are shown in the lower right corner of each actogram.

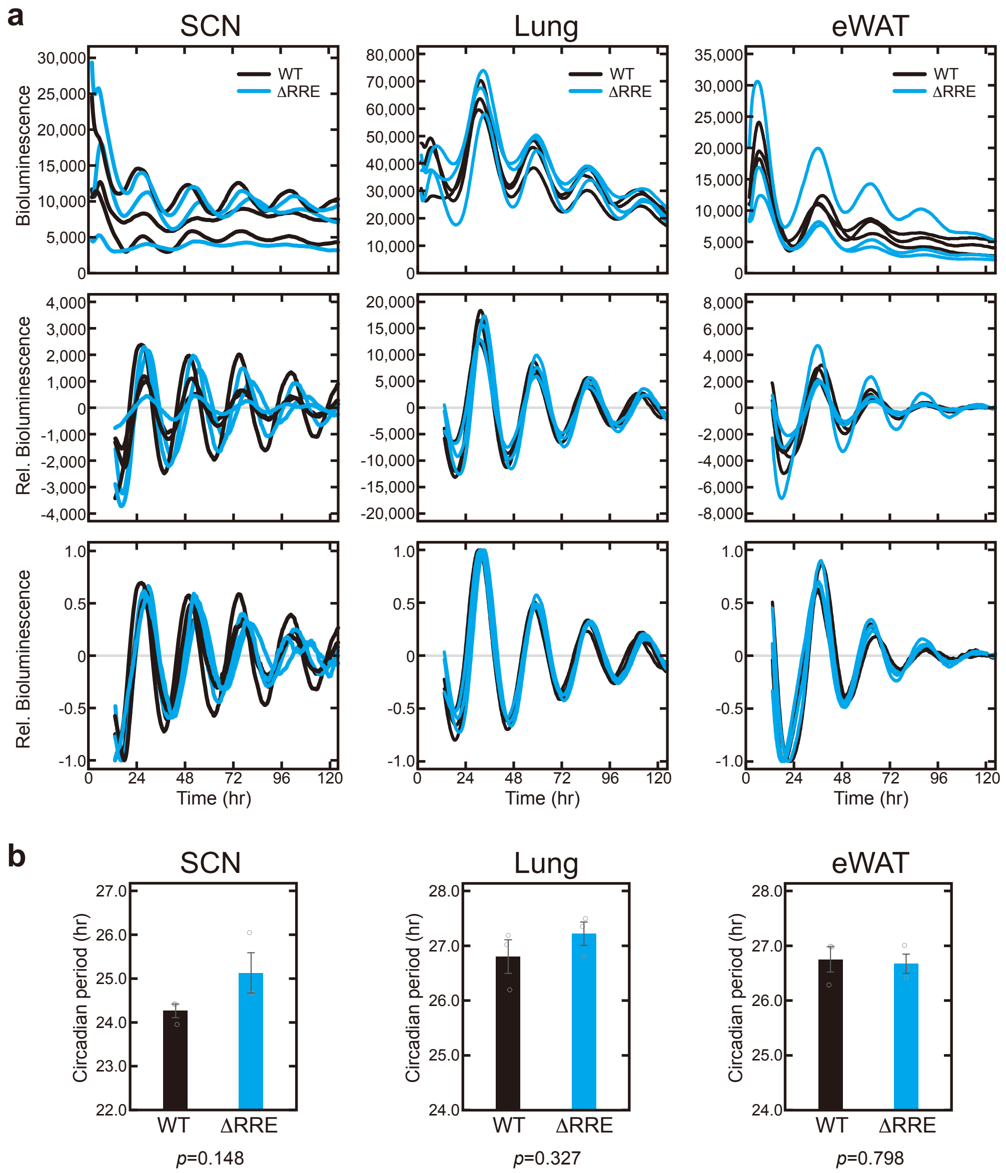

Supplementary Fig. 5.

Bioluminescence rhythms of mouse tissues were maintained in the ΔRRE mutant.

(**a**) Bioluminescence rhythms in the cultured SCN slice, lung and eWat isolated from PER2::LUC-WT and PER2::LUC-ΔRRE mice. The tissues were cultured on the Millicell membrane for the long-term monitoring. The upper, middle, and bottom panels show the bioluminescence data smoothed by 2-hr moving averages, the data detrended by subtracting 24-hr centered moving averages, and the data normalized to the maximum value in the detrended data as 1. The data of ΔRRE mutants and WT (littermate) controls were shown in blue and black , respectively. (**b**) The circadian periods of the rhythms in the SCN, lung and eWAT are shown. Data are means ± SEM (n = 3). Source data are provided as a Source Data file.

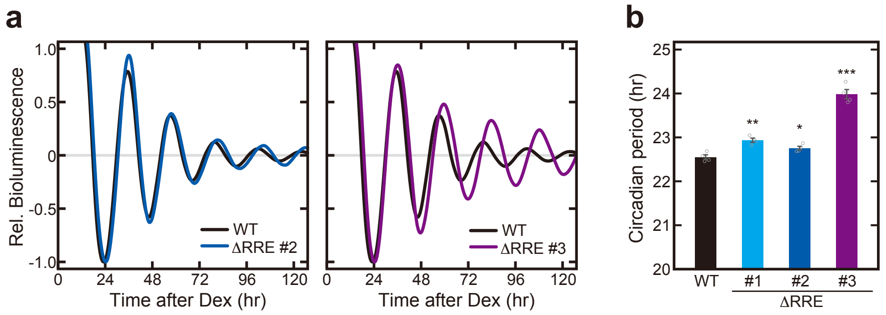

Supplementary Fig. 6.

Cellular bioluminescence rhythms were maintained in the ΔRRE mutant NIH3T3 cells.

(**a**) Representative relative bioluminescence rhythms from the ΔRRE mutant and WT NIH3T3 cells. A Luciferase reporter containing RREs was transiently transfected to monitor the bioluminescent rhythm. Cells were synchronized by 2-hr pulse treatment with dexamethasone just before the monitoring. Traces in dark blue, purple and black indicate the signals recorded from ΔRRE mutant #2 cells, ΔRRE mutant #3 cells and WT cells, respectively. Data of WT cells were reproduced from Fig. 2f. (**b**) The circadian periods of the cellular rhythms. Data of the ΔRRE mutant #1 and WT cells were reproduced from Fig. 2g. Data are means ± SEM (n = 4). Two-sided Student's *t* test, *$P < 0.05$; **$P < 0.01$; ***$P < 0.001$ vs. WT (#1, P=0.0016; #2, P=0.023; #3, P=0.000018). Source data are provided as a Source Data file.

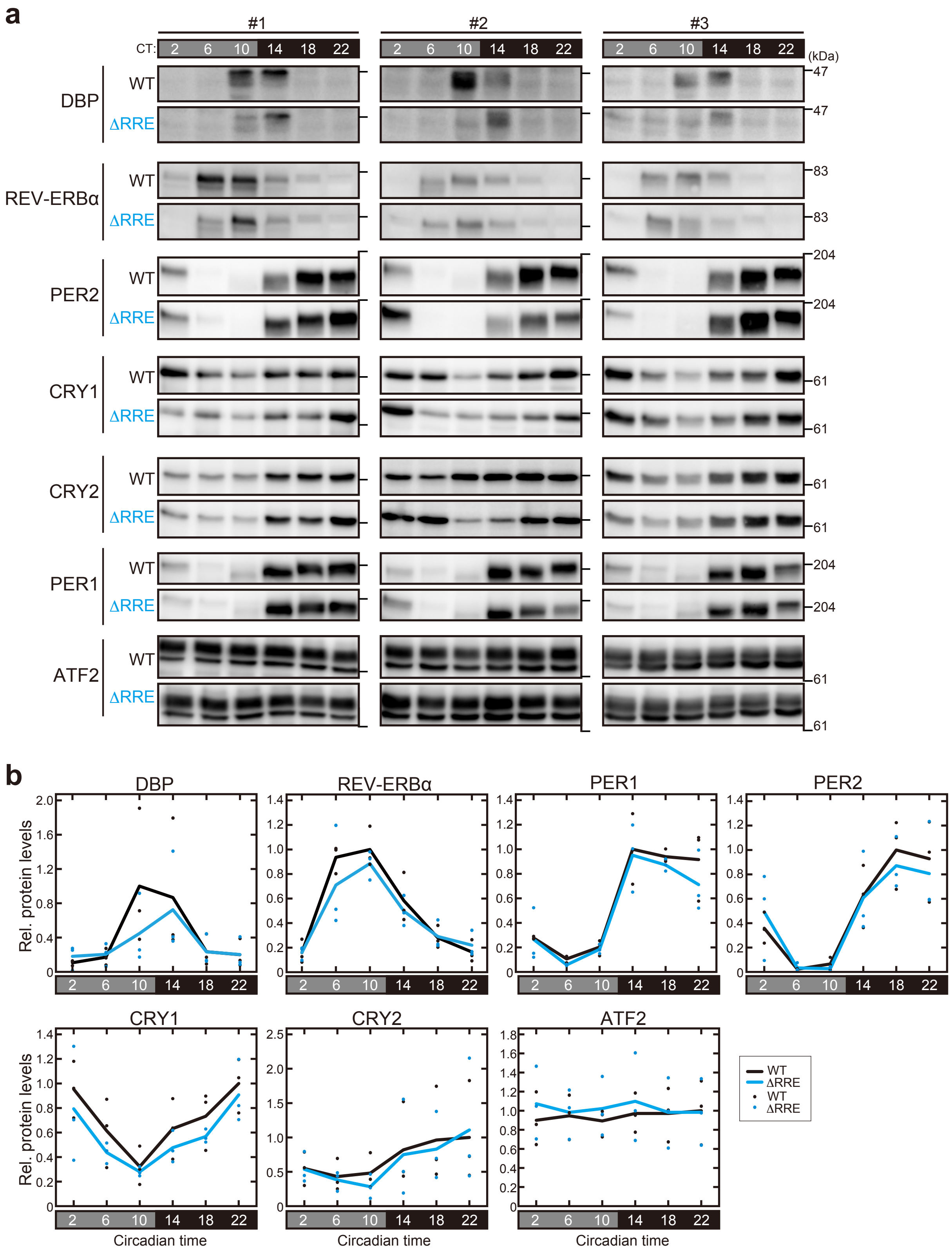

Supplementary Fig. 7.

The rhythmicity of clock proteins levels was maintained in the ΔRRE mutant mouse.

(**a**) Temporal expression profiles of clock proteins in the nuclei of the ΔRRE mutant and WT mouse livers. The mouse liver nuclei prepared at 4-hr intervals were subjected to immunoblot analysis using a series of antibodies for clock proteins. Horizontal grey and black bars above protein bands indicate subjective day and night in the constant-dark (DD) cycle, respectively. (**b**) Quantified data of the immunoblot analysis. Data are represented as dots (n=3) and as lines for means.

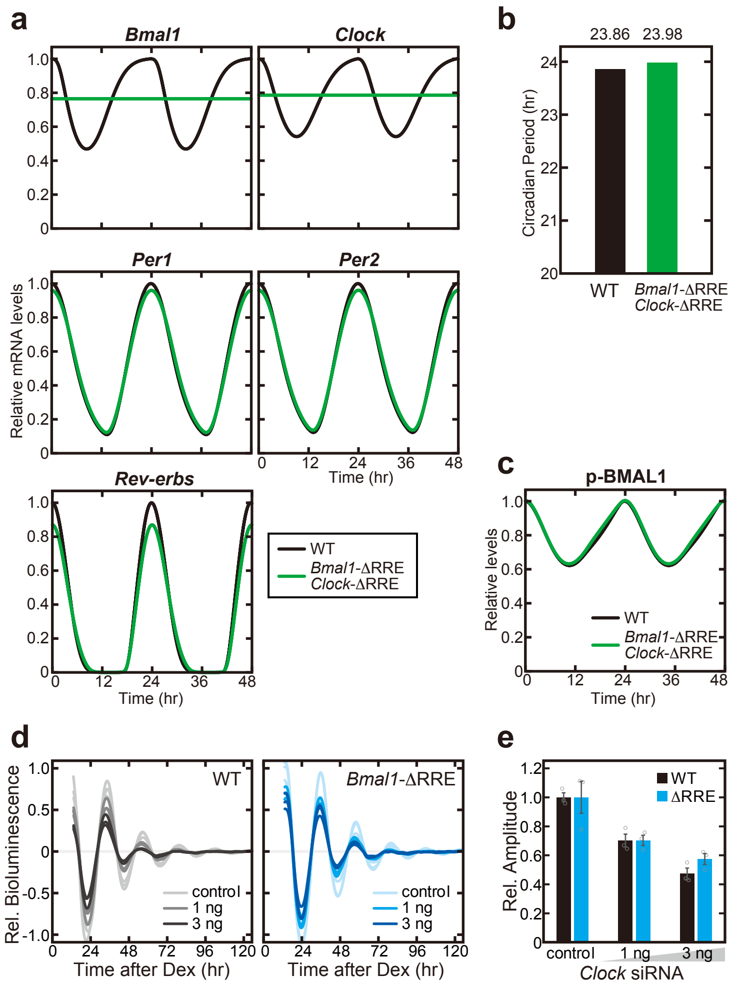

Supplementary Fig. 8.

The circadian oscillation was observed even in the mutant model deficient for the transcriptional rhythms of both *Bmal1* and *Clock*.

(**a**) Simulated temporal mRNA expression profiles of clock genes. Black and Green lines respectively indicate relative mRNA levels in the WT model and the *Bmal1*- and *Clock*-ΔRRE mutant model in which *Bmal1* mRNA and *Clock* mRNA are expressed at their constant levels by the mutations of their RREs. (**b**) Simulated circadian periods in the *Bmal1*- and *Clock*-ΔRRE mutant and WT models. (**c**) Simulated expression profiles of phosphorylated BMAL1 in the *Bmal1*- and *Clock*-ΔRRE mutant and WT models. (**d**-**e**) Effects of CLOCK knockdown on the bioluminescence rhythms in the ΔRRE mutant and WT NIH3T3 cells. Relative amplitudes of the cellular rhythms are shown in (**e**) as means ± SEM (n = 3). Source data are provided as a Source Data file.

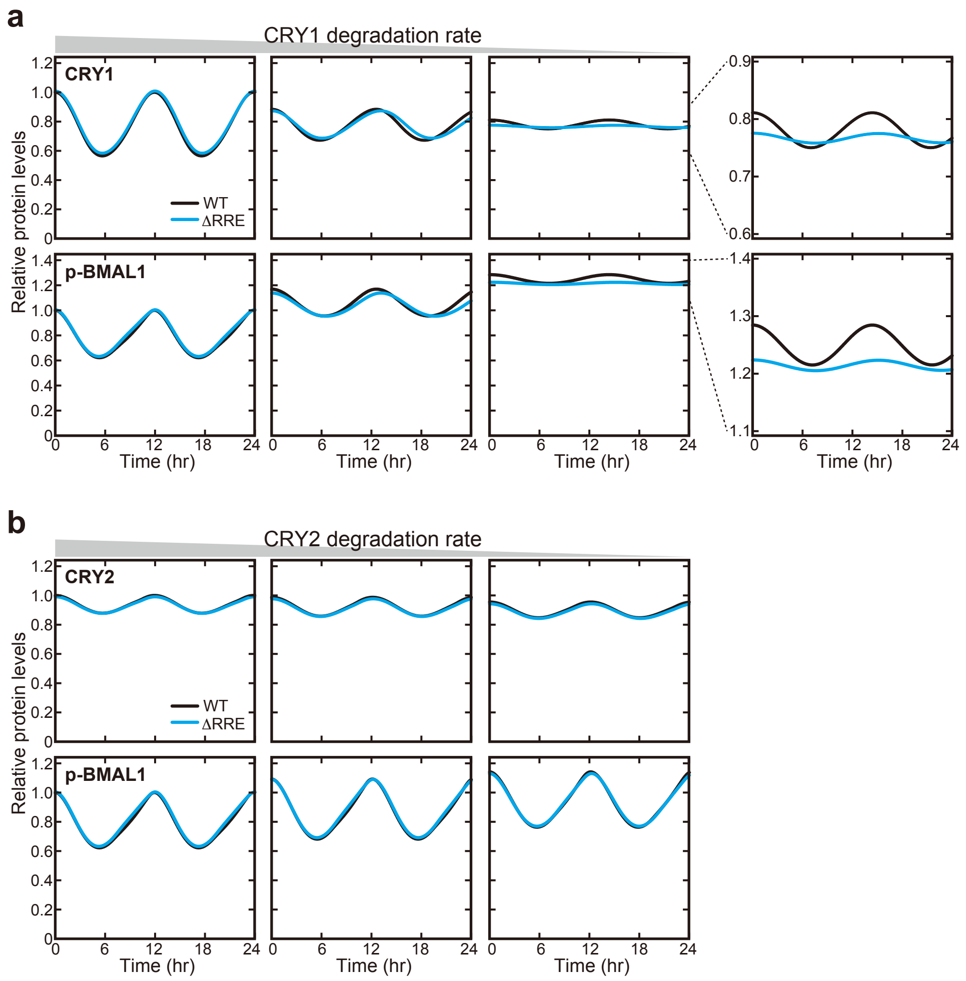

Supplementary Fig. 9.
The perturbation of CRY1 degradation rate had a larger effect on the phospho-BMAL1 rhythm than that of CRY2 degradation rate.

(**a**) Simulated temporal expression profiles of CRY1 (*Upper* panel) and phosphorylated BMAL1 (*Lower*) depending on CRY1 degradation rate. The degradation rate of CRY1 in the nucleus was decreased by 60% (*Center* panel) and 75% (*Right*). Enlarged figures of the *Right* panel are shown next to them. (**b**) Simulated temporal expression profiles of CRY2 (*Upper* panel) and phosphorylated BMAL1 (*Lower*) depending on CRY2 degradation rate. The degradation rate of CRY2 in the nucleus was decreased by 60% (*Center* panel) and 75% (*Right*). Blue and black lines indicate the ΔRRE mutant and WT models, respectively.

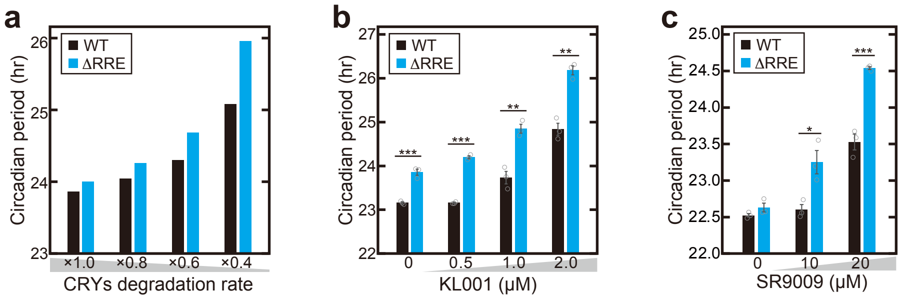

Supplementary Fig. 10.
The circadian period of the ΔRRE mutant is sensitively lengthened by perturbations of CRY1 protein rhythm.

(**a**) Simulated circadian periods in the ΔRRE mutant and WT models when the CRYs degradation rate was decreased (*i.e.*, CRYs were more stabilized) by 20%, 40% and 60%. (**b**) Effects of KL001 on the circadian period of the bioluminescence rhythms in the ΔRRE mutant and WT NIH3T3 cells. Data are means ± SEM (n = 3). Two-sided Student's *t* test, $^{**}P < 0.01$; $^{***}P < 0.001$ vs. WT (0 μM, P=0.00070; 0.5 μM, P=0.0000079; 1.0 μM, P=0.0031; 2.0 μM, P=0.0015). Source data are provided as a Source Data file. (**c**) Effects of SR9009, an agonist of REV-ERBs, on the circadian period of the bioluminescence rhythms in the ΔRRE mutant and WT NIH3T3 cells. Data are means ± SEM (n = 3). Two-sided Student's *t* test, $^{*}P < 0.05$; $^{***}P < 0.001$ vs. WT (10 μM, P=0.020; 20 μM, P=0.00076). Source data are provided as a Source Data file.

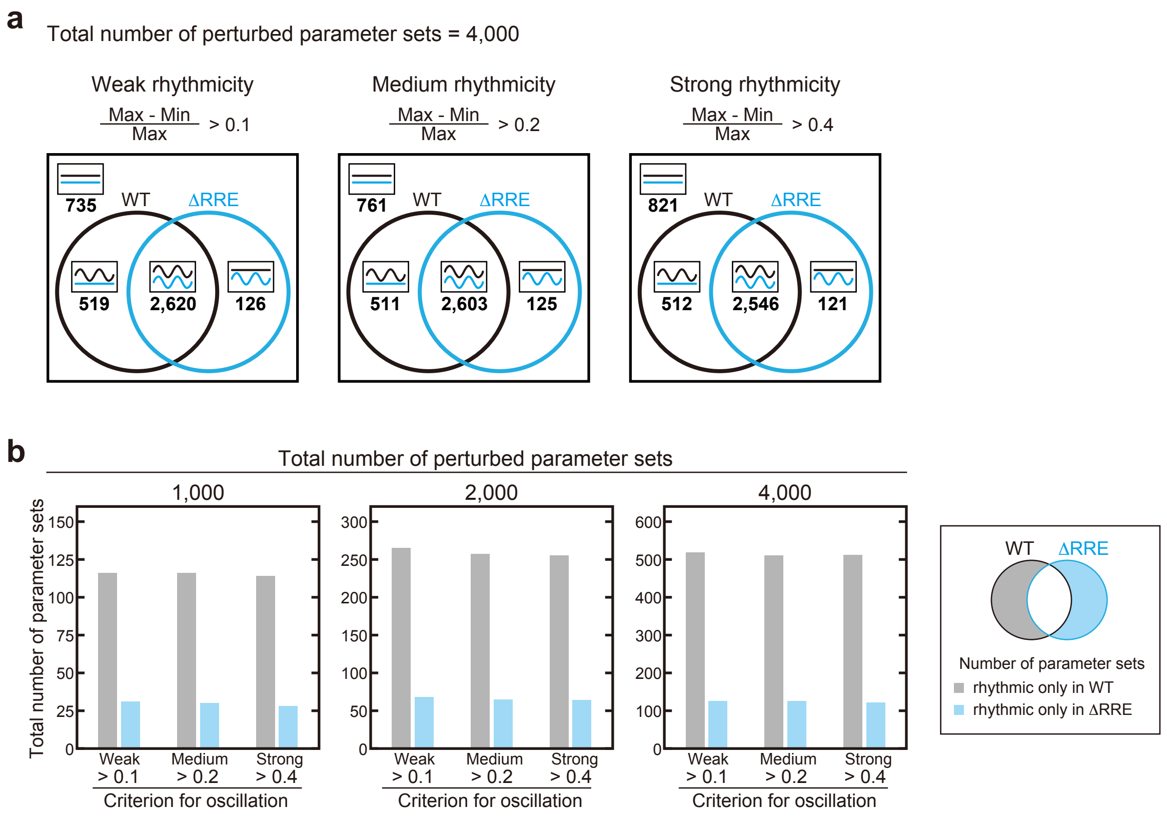

Supplementary Fig. 11.

The WT model is more robust to random parameter variations than the ΔRRE model.

(**a**) Venn diagrams depicting the number of parameter sets that permit oscillations in the ΔRRE mutant and WT models with various criteria for oscillation. Four thousand parameter sets were obtained by randomly perturbing the values of the model parameters within a range of ±50% (see Materials and Methods). (**b**) The number of perturbed parameter sets that permit oscillations only in the WT model (shown in gray) and only in the ΔRRE model (shown in light blue).

a

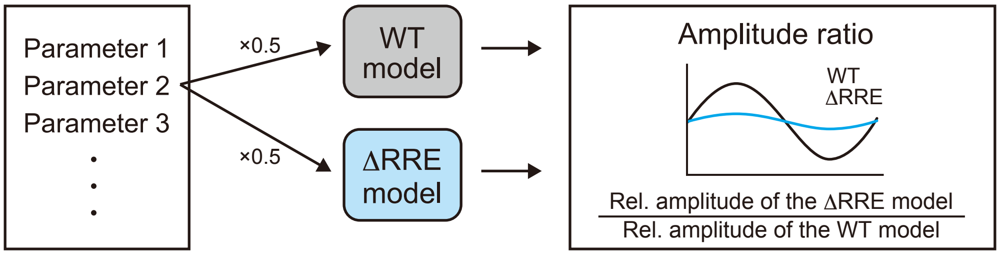

b

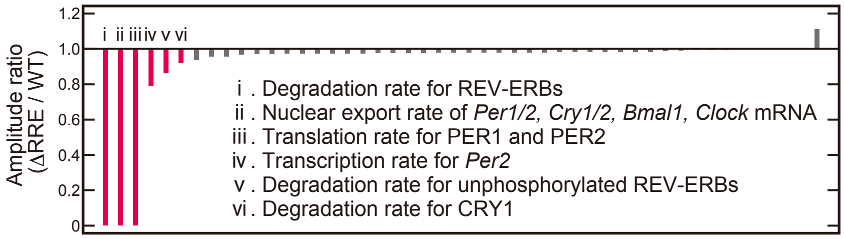

Supplementary Fig. 12.

Perturbation of parameters associated with REV-ERBs and CRYs largely decreased the amplitude in the ΔRRE model.

(**a**) A schematic illustration describing how to estimate the influence of individual parameter perturbation on the circadian rhythms in the two models. Each model parameter was decreased by 50%, and then the effect on the rhythm amplitude in the WT and mutant models was examined. (**b**) Amplitude ratio (ΔRRE/WT) when each parameter was perturbed. Highlighted in red are the top six parameter perturbations that largely decrease amplitude in the ΔRRE model. Source data are provided as a Source Data file.

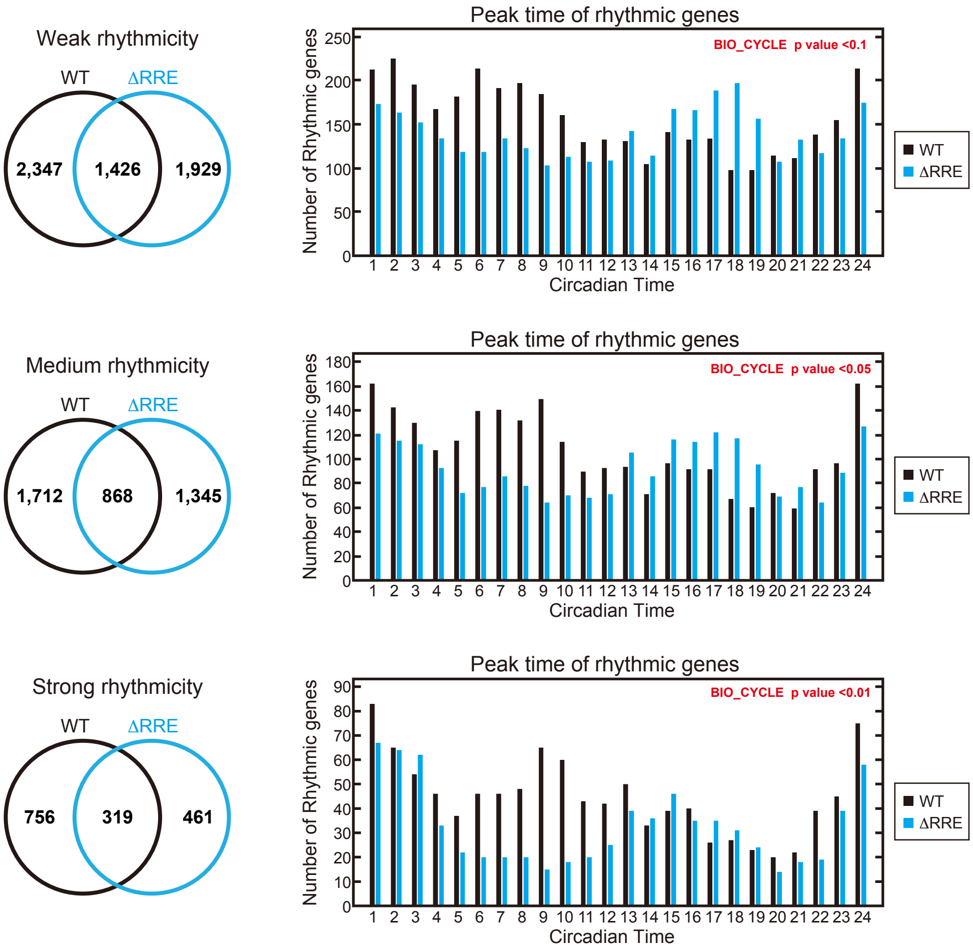

Supplementary Fig. 13.
The number of rhythmic genes peaking in the afternoon was reduced in the ΔRRE mutant mouse liver.

Venn diagram of the number of genes showing weak rhythmicity (*Upper* panel), medium rhythmicity (*Middle*), and strong rhythmicity (*Lower*) in the transcriptome analysis of the ΔRRE mutant and WT mouse liver. Histograms of the number of rhythmic genes peaking at each circadian time were shown in the right panel. Bars in blue and black indicate the gene numbers in the ΔRRE mutant and WT littermate, respectively.

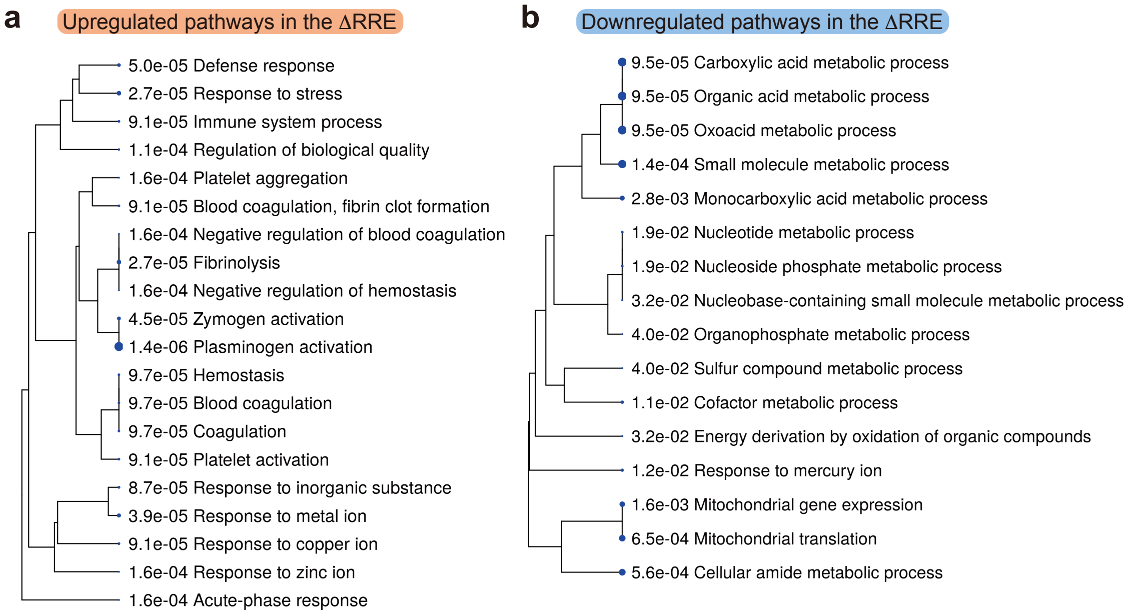

Supplementary Fig. 14.
The total-proteome analysis reveals that the ΔRRE mutation might result in physiological abnormalities.

(**a**,**b**) Hierarchical clustering trees from Gene Ontology (GO) analysis. Among 2,974 proteins that were quantified in the total-proteome analysis across the 24 hours, we identified 66 upregulated proteins and 47 downregulated proteins in the mutant when the mean values of all the time points were compared between the ΔRRE mutant and WT samples (see Dataset S2 for details). The upregulated proteins and the downregulated proteins were functionally categorized by GO analysis, and the top 20 significantly enriched pathways were illustrated in the hierarchical clustering trees. Bigger dots indicate more significant *p* values. The lists of the significant pathways are in Dataset S3.

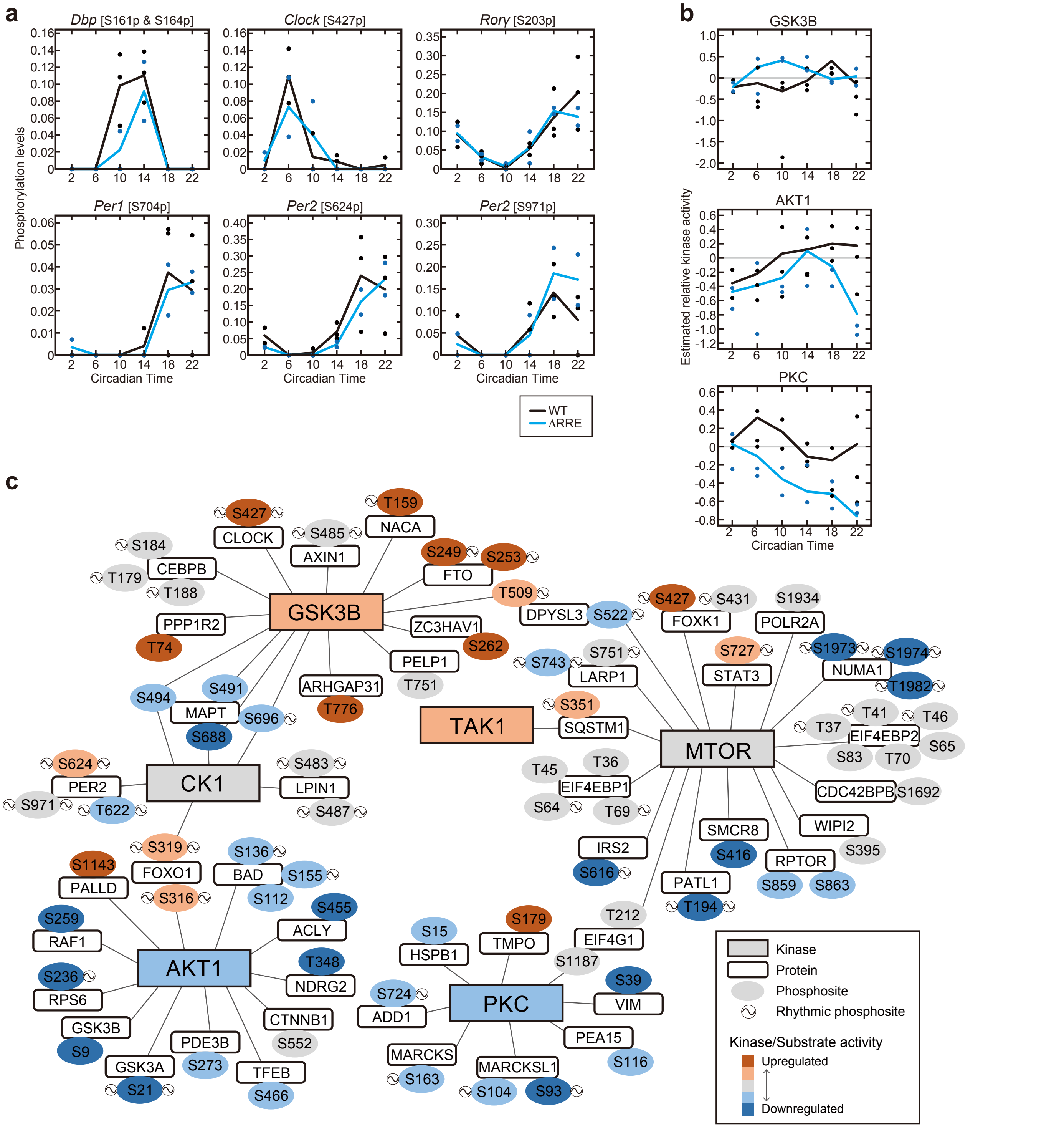

Supplementary Fig. 15.

The phospho-proteome analysis reveals that rhythmicities of some kinase activities and phosphorylated peptides are affected by the ΔRRE mutation.

(**a**) Temporal profiles of representative phosphorylated peptides derived from clock proteins. Phosphorylated peptides (phospho-peptides) were enriched by a FeNTA column and subjected to LC-MS/MS analysis, by which 6,348 phospho-peptides were quantified across the 24 hours (see Dataset S4 for details). Data are represented as dots for individual samples and as lines for means. (**b**) Representative kinase activity profiles estimated by Kinase-Substrate Enrichment Analysis (KSEA) based on kinase-substrate relationships. Positive and negative values indicate relatively higher and lower kinase activities, respectively. (**c**) The network of representative protein kinases and their substrates identified in the phospho-proteome analysis. Kinases/substrates upregulated by the ΔRRE mutation are shown in red, and those downregulated are shown in blue. Rhythmic phosphosite are labeled with a “rhythmic symbol” in WT (at the left side of each phosphosite) or the ΔRRE mutant (at the right side).

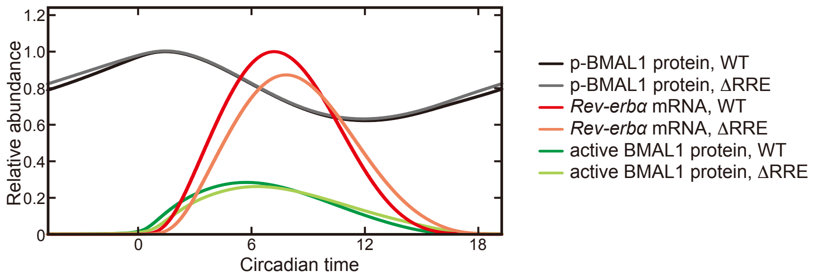

Supplementary Fig. 16.

The phase relationship between the peak times of phospho-BMAL1 rhythm and *Rev-erbα* mRNA rhythm was lengthened by the ΔRRE mutation.

Simulated temporal expression profiles of the phosphorylated BMAL1 protein, *Rev-erbα* mRNA, and active BMAL1 protein. The phosphorylated BMAL1 and active BMAL1 abundances were normalized by the maximum BMAL1 protein abundance in the WT model. The *Rev-erbα* mRNA abundance was normalized by the maximum *Rev-erbα* abundance in the WT model.

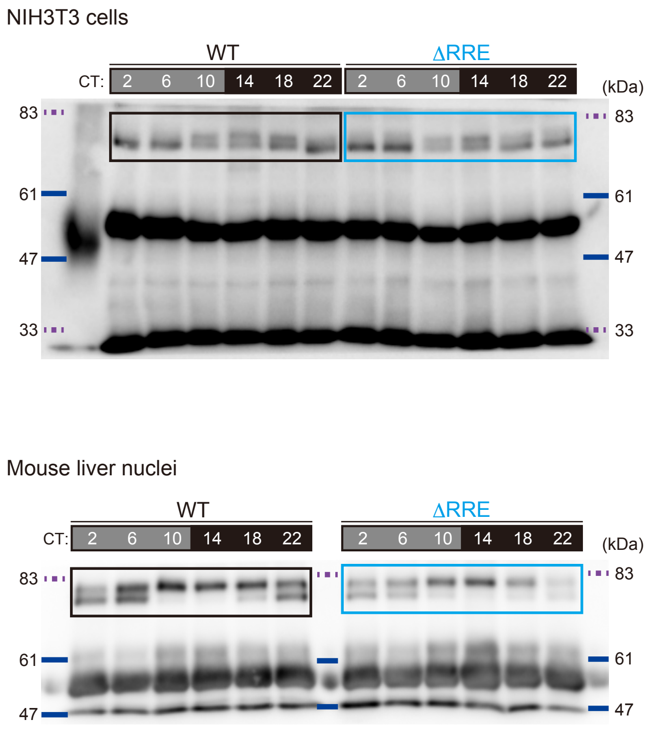

Supplementary Fig. 17.
Uncropped versions of the immunoblot analysis data shown in Fig. 3d.

Circadian variation of BMAL1 phosphorylation in the CLOCK-BMAL1 complex in NIH3T3 cells (*Upper* panel) and mouse liver nuclei (*Lower*). The cropped areas are indicated by black (WT) and blue (ΔRRE) squares.

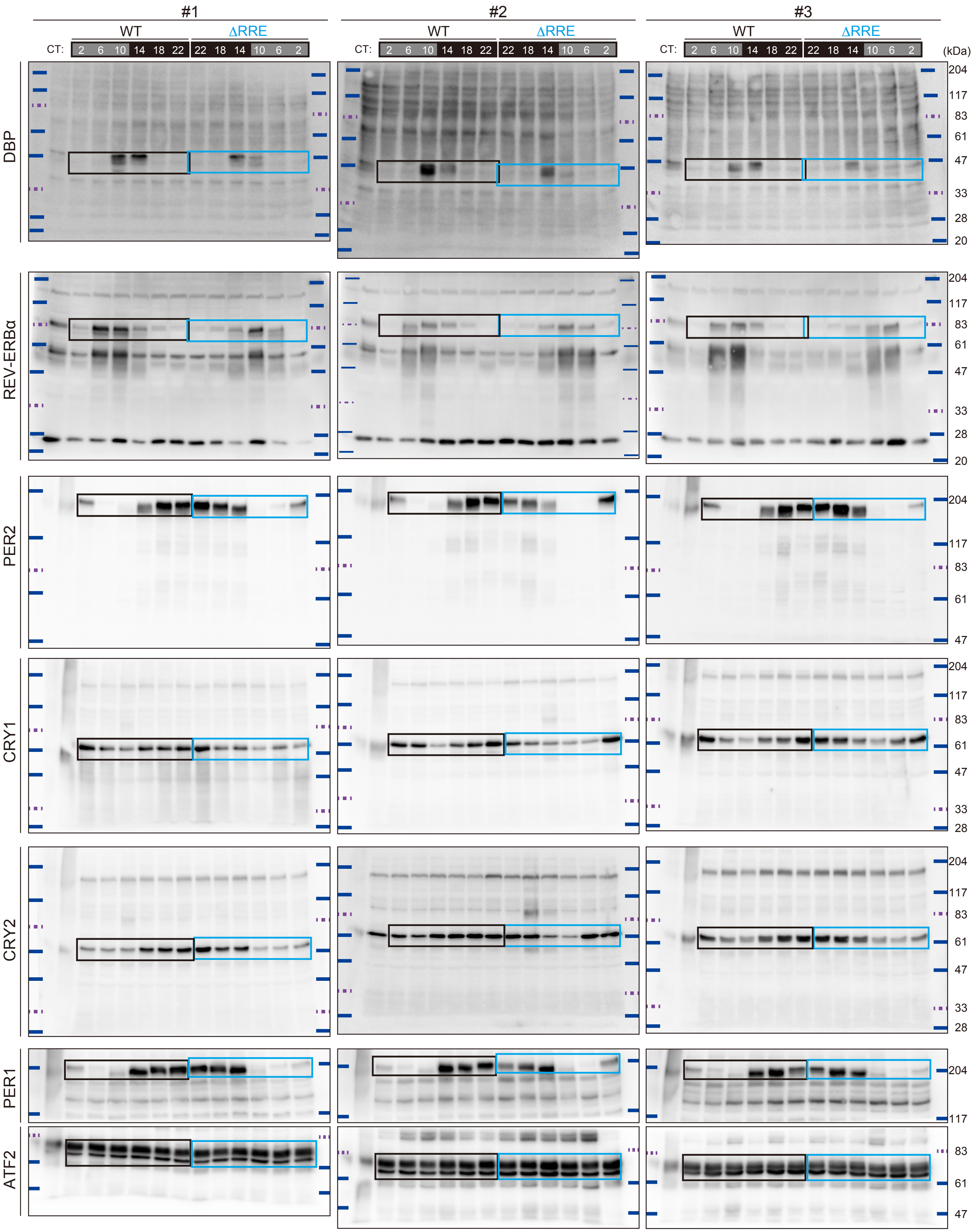

Supplementary Fig. 18.

Uncropped versions of the immunoblot analysis data shown in Supplementary Fig. 7.

Temporal expression profiles of clock proteins in the nuclei of the ΔRRE mutant and WT mouse livers. The cropped areas are indicated by black (WT) and blue (ΔRRE) squares.

Table S1. sgRNA sequences

sgRNA name	Guide sequence
Bmal1-ΔRRE-sgRNA1	GAGCGGATTGGTCGGAAAGT
Bmal1-ΔRRE-sgRNA2	GGGAAGGCAGAAAGTAGGTC
Bmal1-KO-sgRNA1	GTAAACTCACCGTGCTAAGGA
Bmal1-KO-sgRNA2	GAACACGTAACCCTAAGTGAC
Cry1-KO-sgRNA1	GAGCGGATGGTGTCGGCGCCC
Cry1-KO-sgRNA2	GTTGGAAGAGCCGGCGAACC
Cry2-KO-sgRNA	GTCCGCGCCCATCGATTGCG

Table S2. Primer sequences for qRT-PCR

Primer name	Sequence (5' to 3')
Bmal1-Fw	GCAGTGCCACTGACTACCAAGA
Bmal1-Rv	TCCTGGACATTGCATTGCAT
Dbp-Fw	AATGACCTTTGAACCTGATCCCGCT
Dbp-Rv	GCTCCAGTACTTCTCATCCTTCTGT
Rev-erbα-Fw	CGTTCGCATCAATCGCAACC
Rev-erbα-Rv	GATGTGGAGTAGGTGAGGTC
E4bp4-Fw	GTCTTCTGATGGGGAAGACG
E4bp4-Rv	TCCACTGGAGAATGGATGG
Clock-Fw	CCTATCCTACCTTGGCCACACA
Clock-Rv	TCCCGTGGAGCAACCTAGAT
Rps29-Fw	TGAAGGCAAGATGGGTCAC
Rps29-Rv	GCACATGTTCAGCCCGTATT
