# Supplementary material for: Rhythmic transcription of Bmal1 stabilizes the circadian timekeeping system in mammals
Source: Nat Commun. 2022 Aug 23;13:4652. doi: 10.1038/s41467-022-32326-9 (PMC9399252; doi:10.1038/s41467-022-32326-9)
Supplement: Supplementary file 3 — Description of Additional Supplementary Files [file 41467_2022_32326_MOESM3_ESM.pdf]

Title: Supplementary Data 1
Description: Gene expression levels in the wild type and ΔRRE liver. Sheet1, "Transcriptome data": Processed transcriptome data representing FPKM values for each gene. Sheet2, "Mapped reads": Results of the RNA-seq read alignment.

Title: Supplementary Data 2
Description: Semi-quantified amounts of peptides in the total proteome analysis. The amount of each peptide in ΔRRE was averaged with all data points, divided by the averaged value of WT, and shown by a fold change ["Ratio (ΔRRE/WT)"]. Upregulated or downregulated proteins were highlighted as ["UP or DOWN (in ΔRRE)"] according to the analysis of ["Ratio (ΔRRE/WT)"] and two-sided Student's t-test ["TTEST (ΔRRE vs WT)"]. Gene Accessions correspond to SwissProt ID.

Title: Supplementary Data 3
Description: Gene Ontology (GO) enrichment analysis with differentially expressed genes in ΔRRE. Upregulated and downregulated genes were independently subjected to GO analysis. Genes are represented as SwissProt ID.

Title: Supplementary Data 4
Description: Semi-quantified amount of phosphorylated peptides in the phospho-proteome analysis. The circadian rhythmicity of the amount of phosphorylated peptides in the ΔRRE mutant and WT. Rhythmic genes were identified by the BIO_CYCLE and described in the "Rhythm detection" column (see also Methods).

Title: Supplementary Data 5
Description: The time-of-day-dependent changes in kinase activityies in the wild type and ΔRRE mutant. Sheet1, "KSEA_Kinase": A kinase activity score is shown by the log2 fold changes normalized by the total amount of substrate peptides in WT samples. Sheet2, "KSEA_Substrate": The semi-quantified amount of each substrate peptide.
